# Global analysis of the Hfq-mediated RNA interactome discovers a MicA homolog that affects the cytotoxicity, biofilm formation, and resistance to complement of *Bordetella pertussis*

**DOI:** 10.1093/nar/gkaf614

**Published:** 2025-07-08

**Authors:** Dilip Kumar, Martin Beles, Argha Saha, Ilona Procházková, Ana Dienstbier, Jakub Držmíšek, Jan Čapek, Ivana Čurnová, David Hot, Denisa Petráčková, Branislav Večerek

**Affiliations:** Laboratory of post-transcriptional control of gene expression, Institute of Microbiology of the Czech Academy of Sciences, Prague 14200, Czech Republic; Laboratory of post-transcriptional control of gene expression, Institute of Microbiology of the Czech Academy of Sciences, Prague 14200, Czech Republic; Laboratory of post-transcriptional control of gene expression, Institute of Microbiology of the Czech Academy of Sciences, Prague 14200, Czech Republic; Laboratory of post-transcriptional control of gene expression, Institute of Microbiology of the Czech Academy of Sciences, Prague 14200, Czech Republic; Laboratory of post-transcriptional control of gene expression, Institute of Microbiology of the Czech Academy of Sciences, Prague 14200, Czech Republic; Laboratory of post-transcriptional control of gene expression, Institute of Microbiology of the Czech Academy of Sciences, Prague 14200, Czech Republic; Laboratory of post-transcriptional control of gene expression, Institute of Microbiology of the Czech Academy of Sciences, Prague 14200, Czech Republic; Laboratory of post-transcriptional control of gene expression, Institute of Microbiology of the Czech Academy of Sciences, Prague 14200, Czech Republic; University of Lille, CNRS, Inserm, CHU Lille, Institute Pasteur de Lille, US 41 - UAR 2014 - PLBS, F-59000Lille, France; Laboratory of post-transcriptional control of gene expression, Institute of Microbiology of the Czech Academy of Sciences, Prague 14200, Czech Republic; Laboratory of post-transcriptional control of gene expression, Institute of Microbiology of the Czech Academy of Sciences, Prague 14200, Czech Republic

## Abstract

*Bordetella pertussis* is a Gram-negative, strictly human re-emerging respiratory pathogen and the causative agent of whooping cough. The requirement of the RNA chaperone Hfq for the virulence of *B. pertussis* suggests that Hfq-dependent small regulatory RNAs (sRNAs) are involved in the virulence of this pathogen. To identify their potential mRNA targets, we applied a method combining experimental and computational approaches called RIL-seq. The majority of putative mRNA targets, including several virulence factors, interact with two sRNAs, CT_433 and CT_521, suggesting that these sRNAs may represent central riboregulatory nodes of *B. pertussis*. Furthermore, our data suggest that CT_532 sRNA can base pair with the 5′UTR region of *ompA* mRNA encoding outer membrane protein BP0943 (OmpA) and that CT_532, RNase III and Hfq are involved in the control of *ompA* expression. The CT_532 sRNA shares 60% identity with the *E. coli* sRNA MicA and its expression is also modulated by Hfq and stress conditions such as heat and cold shocks. Overall, these results suggest that CT_532 represents a MicA homolog. Importantly, the mutant lacking the first 22 nucleotides of CT_532 exhibits reduced cytotoxicity towards human macrophages and impaired biofilm production but increased resistance to complement compared to the wild type strain.

## Graphical Abstract

**Graphical Abstract:**
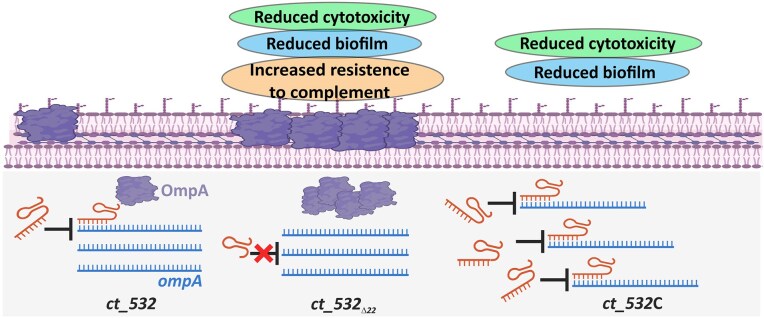

## Introduction

*Bordetella pertussis* is a Gram-negative strictly human pathogen of the respiratory tract and the etiological agent of whooping cough (pertussis) [1]. This highly contagious disease is particularly severe in infants and remains a major cause of infant mortality and morbidity worldwide, especially in developing countries [2]. Furthermore, pertussis incidence is currently on the rise in industrialized countries with highly vaccinated populations [3] and since 2023, several countries experienced epidemics of pertussis [4]. The global resurgence of pertussis suggests that we need to expand our understanding of the molecular mechanisms underlying the pathogenesis of *B. pertussis*, including post-transcriptional regulation. In many pathogenic bacteria, the RNA chaperone Hfq and small regulatory RNAs (sRNAs) emerged as critical players in the post-transcriptional control of gene expression [5, 6]. Based on their chromosomal location in regard to their targets, sRNAs are divided into two groups: *cis*- and *trans*-encoded. The *cis*-encoded antisense RNAs (asRNAs) are transcribed antisense to their targets and therefore exhibit perfect complementarity with the regulated mRNAs [7, 8]. In contrast, *trans*-encoded sRNAs are transcribed from genomic loci other than their target genes and exhibit only partial complementarity with the target transcripts. In Gram-negative bacteria, post-transcriptional regulation based on imperfect sRNA/mRNA duplexes often requires the activity of RNA chaperones such as Hfq [9, 10]. The Hfq protein forms ring-shaped hexamers that possess several RNA-binding sites that allow simultaneous interaction with both sRNA and mRNA molecules and stabilization of their interactions [9, 11, 12]. Besides its role in facilitation and stabilization of RNA duplexes, Hfq can actively remodel the structure of RNAs and also increase the stability of sRNAs [9, 10]. Importantly, Hfq-associated sRNAs have been shown to be involved in a variety of cellular processes, such as iron homeostasis, membrane remodeling, energy metabolism, and quorum sensing [6, 13–16]. Given that a pathogenic lifestyle often requires a swift response to rapid changes in environmental conditions, it is not surprising that a large number of sRNAs produced by bacterial pathogens are involved in stress adaptation and virulence [5, 14].

Previously, the role of post-transcriptional regulation in the physiological fitness and virulence of *B. pertussis* has been studied only sporadically. An initial attempt to identify sRNAs in this pathogen was made by Hot and colleagues in 2011 [17]. Using bioinformatics, they identified 23 sRNAs, 13 of which were experimentally validated. Over the past decade, we have, in collaboration with our colleagues, pioneered the field of post-transcriptional control of gene expression in *B. pertussis*. First, we showed that the RNA chaperone Hfq is required for virulence [18] and T3SS functionality [19], and then we determined the global Hfq regulon both on transcriptomic and proteomic levels [20]. Next, we have determined the primary transcriptome of *B. pertussis* and annotated hundreds of putative non-coding RNAs referred to as Candidate_Transcripts (CT) [21]. This study has also revealed that Insertion Sequence (IS) elements such as IS*481*, present in more than 240 copies in the *B. pertussis* genome, impact on transcriptional landscape of this pathogen. Furthermore, we characterized the first non-coding regulatory RNA of *B. pertussis*, RgtA, and showed that its expression is dependent on the system of signal transduction BvgAS [22]. The BvgAS system consists of the sensor kinase BvgS and the response regulator BvgA and promotes the expression of virulence-activated genes [23]. Under *in vitro* conditions, three different phenotypes can be distinguished based on the BvgA phosphorylation status and the presence and concentration of the modulators magnesium sulfate and nicotinate: virulent Bvg^+^ (no modulators), intermediate Bvg^i^ (10–20 mM sulfate or 5–10 mM nicotinate), and avirulent Bvg^−^ (50 mM sulfate or 20 mM nicotinate) [24, 25].

Finally, our dual RNA-seq analysis of infected human macrophages revealed that the levels of several non-coding RNAs were significantly modulated in intracellular *B. pertussis* cells [26]. In the meantime, other groups have begun to explore non-coding RNAs in *B. pertussis*, including those regulated by the BvgAS system [27–29]. Collectively, these data suggest that non-coding sRNAs play an important role in the virulence and physiology of *B. pertussis* and thus, could serve as potential targets for novel therapeutic interventions.

With the advancement of RNA-seq technology, hundreds of sRNAs have been discovered in various bacterial genomes, although the function of most sRNAs remained uncharacterized [30]. Indeed, identification of mRNA targets that are directly regulated by sRNAs has been the rate-limiting step in their functional characterization. *In silico* prediction of transcripts targeted by *trans*-encoded regulatory sRNAs is not trivial for several reasons, such as limited and imperfect base pairing with the target mRNA, and requires strenuous experimental validation. High-throughput transcriptomics made it possible to determine global responses to overexpression or deletion of the analyzed sRNA, but usually only a single sRNA could be examined at a time. Recently, a method combining experimental and computational approaches called RIL-seq (RNA interaction by ligation and sequencing) has been developed [31, 32] and successfully used in several studies [33–38]. It is based on immunoprecipitation of *in vivo* formed Hfq-RNA complexes, *in vitro* ligation of the RNAs bound to Hfq, sequencing of the ligated chimeric RNAs and computational analysis. This method thus enables the global capture and mapping of *in vivo*-formed duplexes between sRNAs and target mRNAs.

In this study, we used the RIL-seq method to identify Hfq-mediated RNA-RNA interactions in exponentially grown *B. pertussis* cells. We identified plausible targets for several previously identified sRNAs, including CT_532 sRNA, a likely homolog of the *E. coli* sRNA MicA. We analyzed the expression profiles of CT_532 and characterized the phenotypic traits of the mutant carrying deletion in the *ct_532* locus.

## Materials and methods

### Bacterial strains and growth conditions

*Bordetella* strains and their derivatives listed in Supplementary Table S1 were grown on Bordet Gengou agar (BGA, Difco) supplemented with 15% defibrinated sheep blood at 37°C. For planktonic cultures, bacteria were grown in Stainer-Scholte (SS) medium supplemented with 0.1% cyclodextrin and 0.5% casamino acids at 37°C. To harvest samples for RIL-seq experiment, five independent cultivations of Tohama I strain carrying wild type (wt) copy of the *hfq* gene (Hfq-WT) and its isogenic mutant carrying ain-frame *hfq-3xFLAG* fusion (Hfq-FLAG) were performed to collect five biological replicates of each strain. Cultures were grown overnight in SS medium to mid exponential phase of growth (OD_600_ ≈ 1.1–1.3). *Escherichia coli* strains used for cloning and conjugation were cultured on Luria-Bertani (LB) agar or in LB broth at 37°C.

### Construction of the mutants

The mutations were introduced into the chromosome of the *B. pertussis* Tohama I strain as described previously [18]. To create a chromosomal *hfq*-3x*FLAG* tag *C*-terminal in-frame fusion, two DNA fragments of approximately 700 bp, flanking the upstream and downstream regions of the TAA stop codon of the *hfq* gene (*bp2193*) and carrying a FLAG tag sequence at 3′ and 5′ end, respectively, were amplified and ligated via a *Psi*I site naturally present in the 3xFLAG sequence. The ligation mixture was used as a template to generate a PCR product in which the 3xFLAG tag was inserted in-frame upstream of the TAA stop codon of the *hfq* gene.

A similar approach was used to construct the deletion mutants. The upstream and downstream DNA fragments flanking the intended deletion carried a *Nhe*I restriction site at the 3′ and 5′ ends, respectively, were cleaved and ligated over the *Nhe*I site. The ligation mixture was used to amplify the PCR product in which we deleted the first 22 nucleotides (nt) of CT_532 sRNA (*ct_532_Δ22_* mutant), 12 bp corresponding to −10 promoter region of CT_521 (−13 to −2 region relative to the 5′ end of the transcript, *ct521_ΔP_* mutant), the sequence corresponding to amino acid residues 2–85 of RNase III (*rnc_Δ85_* mutant), the sequence encoding the *C*-terminal domain of RNase E (residues 579 to 1042, *rne*_*Δ*_*_CTD_* mutant) and the complete *rseA* sequence encoding the anti-sigma factor RseA, which inactivates RpoE (Δ*rseA* mutant).

For all constructs, the final PCR products were ligated into the allelic exchange plasmid pSS4245 and cloned in *E. coli* TXL-Blue cells [39]. The resulting recombinant plasmids were transformed into the *E. coli* SM10 λ-pir strain and transferred into the *B. pertussis* Tohama I strain by conjugation. After two recombination events, the strain carrying the desired mutation was obtained and verified by sequencing of the amplified PCR product covering the adjacent chromosomal regions.

To obtain a strain in which the *ct_532* deletion is complemented, the 193-bp fragment containing the *ct_532* allele, including the promoter and terminator sequence, was amplified by PCR using the forward and reverse primers flanking the *ct_532* allele. The blunt-ended PCR product was inserted into the *Eco*RV site of the cloning vector pBBR1MCS [40]. The resulting plasmid, pBBRCT_532, was checked for the insertion of the *ct_532* gene by sequencing and then transferred into *E. coli* SM10 cells by transformation. Finally, the plasmid was transferred into the *B. pertussis ct_532_Δ_*_22_ strain by conjugation, yielding the *ct_532*C strain. The primers used in this study are listed in Supplementary Table S2.

### RIL-seq and DE analyses

The RIL-seq procedure was performed essentially as described by Melamed *et al.* [31]. Briefly, wt and *hfq-3xFLAG* strains were grown in pentaplicates in SS medium to log phase. Next, cells (10 mL) were pipetted into a Petri dish and exposed to UV irradiation (800 mJ of 254 nm UV) to crosslink the complexes between Hfq protein and RNA. Cells were pelleted in ice cold PBS and mechanically lysed in lysis buffer (50 mM sodium phosphate, 300 mM NaCl, 0.1% IGEPAL, and 10 mM imidazole) supplemented with a protease inhibitor cocktail (5 μL/mL) and RNase inhibitor (100 U/mL) using 400 μL zirconium/glass beads in a FastPrep beater (Thermo Savant). The Hfq-RNA complexes in lysates were coimmunoprecipitated with anti-FLAG antibodies conjugated with M2 magnetic beads (Sigma-Aldrich). The beads were treated with RNase A/T1 mix (Thermo Fisher Scientific) for 5 min at 22°C to trim the bound RNA molecules and then the cleavage was stopped with SUPERase IN RNase inhibitor (Thermo Fisher Scientific). Trimmed ends were modified with polynucleotide kinase (NEB) for 2 h at 22°C with gentle agitation and the Hfq-bound RNA duplexes were ligated with T4 RNA ligase I enzyme (NEB) overnight at 22°C. The ligated RNA was released from Hfq with a proteinase K (ThermoFisher Scientific) digestion for 2 h at 55°C followed by extraction with TRI Reagent LS (Merck). Purified RNA was processed at sequencing facility (Institute of Applied Biotechnologies; https://www.iabio.eu/) where the quality of RNA was assessed using the Agilent RNA 6000 Pico chip on the Bioanalyzer 2100 instrument. The libraries were prepared with SMARTer Stranded Total RNA-seq kit v2-Pico Input (Takara) and sequenced on a NovaSeq 6000 platform (Illumina) using the paired-end 151 base-pair read protocol yielding ≈ 20 million reads per sample). The RNA-seq data used for RIL-seq and DE-seq analyses is deposited at the European Nucleotide Archive under project accession number PRJEB79241. Quality control of the obtained reads was done using FastQC (https://www.bioinformatics.babraham.ac.uk/projects/fastqc/). Quality trimming and adaptor removal from the resulting reads was done using Cutadapt program [41].

RIL-seq computational analysis was conducted as detailed in Melamed *et al.* [31]. Shortly, after pre-processing with Cutadapt, reads were mapped to *B. pertussis* Tohama I reference genome using map_single_fragments.py script with -r parameter (based on Burrows-Wheeler aligner with aln option). In each replicate the mapped fragments were classified as single or chimeric using map_chimeric_fragments.py script. After testing the reproducibility, the replicates were unified and statistically significant chimeras (S-chimeras) were determined using RILseq_significant_regions.py script with Bonferroni correction for multiple hypothesis testing. As a reference, *B. pertussis* Tohama I BioCyc database (BPER257313CYC, version 23.0) [42] was used. Candidate transcripts and other known sRNAs were added to the database manually.

To quantify the transcripts that were enriched in the *hfq-3xFLAG* samples, we performed the differential gene expression (DE) analysis. The reads were mapped to *B. pertussis* Tohama I reference genome using Burrows-Wheeler aligner with mem option (bwa-mem) [43] and the reads per gene counts were calculated using htseq-count program with default parameters [44]. Prior to DE analysis, unwanted variation in the samples caused by batch effects was removed using the RUVs correction method of RUVseq [45]. DE analysis was performed with DESeq2 [46]. Genes with a |log_2_ fold change| > 1 and adjusted *P*-value < 0.05 were considered as significantly deregulated.

### Identification of common binding motifs in target mRNAs

Motifs were searched in each sRNA target sequence set by MEME [47] allowing motif width to range from 6 to 15 nucleotides. The search for a common motif was applied to CT_433 and CT_521 sRNA target sets. The sequences to which the search was applied were extracted from S-chimera coordinates and padded by 50 nt on both sides. To verify that an identified motif matches a complementary binding site on the respective sRNA, we searched for it in the reverse-complement sequence of the sRNA using MAST [48]. We submitted to MAST the motifs found by MEME with an E-value ≤ 10.

### Label-free proteomics by nLC-MS/MS and data analysis

Cultures of *B. pertussis* were pelleted by centrifugation (10 000 g, 4°C, 10 min) to separate cell pellets and culture supernatants. Cells were resuspended in TEAB digestion buffer (100 mM Triethylammonium bicarbonate, pH 8.5, 2% sodium deoxycholate) and lysed by sonication. Protein concentrations were determined using the BCA protein assay kit (Thermo Fischer Scientific) and 30 μg of protein per sample were used for protein analysis. Samples were digested with trypsin (trypsin to protein ration 1:30) reconstituted in 100 mM TEAB and incubated at 37°C overnight. After digestion samples were acidified with trifluoroacetic acid (Sigma) to 1% final concentration and peptides were desalted with C18 disks (Empore) as described [49].

Nano reversed phase columns (Aurora Ultimate TS, 25 cm x 75 μm ID, 1.7 μm particle size, Ion Opticks) were used for LC/MS analysis. Mobile phase buffer A was composed of 0.1% formic acid in water. Mobile phase B was composed of 0.1% formic acid in acetonitrile. Samples were loaded onto the trap column (C18 PepMap100, 5 μm particle size, 300 μm x 5 mm, Thermo Scientific) for 4 min at 18 μL/min. Loading buffer was composed of water, 2% acetonitrile and 0.1% trifluoroacetic acid. Peptides were eluted with Mobile phase B gradient from 4% to 35% B in 60 min. Eluting peptide cations were converted to gas-phase ions by electrospray ionization and analyzed on a Thermo Orbitrap Ascend (Thermo Scientific) by data independent approach. Survey scans of peptide precursors from 350 to 1400 m/z were performed in orbitrap at 60K resolution (at 200 m/z) with a 4 × 10^5^ ion count target. DIA scans were performed in orbitrap at 30K resolution. AGC target was set to 1000% and maximum injection time mode to Auto. Precursor mass range 400–1000 m/z was covered by 30 windows 20 Da wide. Activation type was set to HCD with 28% collision energy.

All data were analyzed and quantified with the Spectronaut 18 software [50] using directDIA analysis. Data were searched against *B. pertussis* strain Tohama database (downloaded from Uniprot.org in March 2024 containing 3 258 entries). Enzyme specificity was set as *C*-terminal to Arg and Lys, also allowing cleavage at proline bonds and a maximum of two missed cleavages. Carbamidomethylation of cysteines was set as fixed modification and N- terminal protein acetylation and methionine oxidation as variable modifications. FDR was set to 1% for PSM, peptide and protein. Quantification was performed on MS2 level. Precursor PEP cutoff and precursor and protein cutoff was set to 0.01, protein PEP was set to 0.05. Data were exported and data analysis was performed using Perseus 1.6.15.0 [51].

### Western blot analysis

Samples of pelleted cells equivalent to 0.1 OD_600_ unit or samples of secreted proteins precipitated from culture supernatants equivalent to 1.0 OD_600_ unit were separated on 12.5% SDS-polyacrylamide gels and transferred onto a nitrocellulose membrane using the Trans-Blot® Turbo™ system (Bio-Rad). Membranes were blocked with 5% skim milk and probed with in-house produced polyclonal antibodies raised against PT_S1_, (1:10 000) in mouse or against FHA (1:500) and TcfA (1:3 000) in rabbit, followed by incubation with anti-mouse IgG or anti-rabbit IgG antibodies conjugated with horseradish peroxidase (Cell Signalling Technology, Inc.) at a 1:10 000 dilution. The antibody-antigen complexes were visualized using SuperSignal West Femto chemiluminescent substrate (Thermo) according to the standard protocol on the G:Box Chemi XRQ device (Syngene).

### Quantification of the RgtA levels

Triplicates of wt and *hfq-3xFLAG* strains were grown in 20 mL of SS medium to log phase. Subsequently, half (10 mL) of cells were lysed directly in TE buffer (10 mM Tris, 1 mM EDTA; pH 8.0) containing 1 mg/mL lysozyme (Sigma-Aldrich) and total RNA was isolated from lysed cells using TRI reagent (Sigma-Aldrich) according to the manufacturer's protocol. Cells from the second half of the culture were UV-crosslinked, pelleted in PBS and lysed using zirconium/glass beads in wash buffer (50 mM sodium phosphate, 300 mM NaCl, 0.1% IGEPAL, and 10 mM imidazole). Then, Hfq-RNA complexes in lysates were coimmunoprecipitated with M2 magnetic beads conjugated with anti-FLAG antibodies (Sigma-Aldrich). The RNA was released from Hfq with a proteinase K (Thermo Fisher Scientific) digestion for 2 h at 55°C followed by extraction with TRI Reagent LS (Merck). The determination of RgtA levels was performed by quantitative PCR (see below).

### Quantitative PCR (RT-qPCR)

Total RNA was isolated from analyzed cells with TRI reagent. DNA was removed by treatment of samples with Turbo DNase kit (Sigma-Aldrich) for 60 min at 37°C. RNA concentration was measured for each sample in technical triplicates using Nanodrop 2000 instrument (Thermo Fisher Scientific). The RNA (1 μg) was reverse transcribed into cDNA using mixture of oligo(dT) and random hexamers and M-MLV reverse transcriptase (Promega) at 37°C in a 25-μL reaction according to manufacturer's instruction. RT-qPCR reactions were performed in at least three technical replicates per sample on Bio-Rad CFX96 instrument using SYBR® Green JumpStart™ Taq ReadyMix™ (Sigma-Aldrich), 4 pmoles of each primer and 2 μL of 30 times diluted cDNA in a 20-μL reaction. Each reaction consisted of an initial step at 95°C for 2 min, 40 cycles of 95°C for 15 s, 62°C for 20 s, and 72°C for 30 s, followed by melting curve recording. The *rpoB* gene was used as the reference gene and relative gene expression was determined using delta-delta C_t_ method [52]. The sequences of primers used for RT-qPCR are listed in the Supplementary Table S2.

### Northern blot analysis

Pellets of *B. pertussis* cells were suspended in TE buffer (10 mM Tris, 1 mM EDTA; pH 8.0) containing 1 mg/ml of lysozyme (Sigma) and total RNA was isolated from lysed cells using TRI Reagent (Sigma) according to manufacturer's protocol. Removal of DNA was achieved by treatment of samples with TURBO DNA-free kit (ThermoFisher Scientific). RNA quality and quantity was determined by agarose gel electrophoresis and using the Nanodrop One machine (ThermoFisher Scientific). For Northern blot analysis, 5 μg of total RNA were mixed with 2x RNA loading dye, heated for 5 min at 65°C before electrophoretic separation on a 8% polyacrylamide-8 M urea denaturing gel prepared in 0.5 × TBE buffer. Biotinylated RNAs transcribed *in vitro* using RNA Century™-Plus Marker Template (Invitrogen) served as size markers. The RNA was then transferred onto a Zeta-Probe nylon membrane (Biorad) by electroblotting and UV-crosslinked. The membrane was hybridized overnight with a biotinylated sRNA-specific probe or after rehybridization with biotinylated 5S rRNA-specific probe 5S_NB. Signals obtained with 5S-specific probe served as loading controls. Blots were developed using chemiluminescent detection with the BrightStar® BioDetect™ kit (Ambion). Hybridization signals were visualized using a G:Box Chemi XRQ device (Syngene). The sequences of biotinylated probes used for Northern blot analysis are listed in the Supplementary Table S2.

### Cytotoxicity of *B. pertussis* towards THP-1 macrophages

Cytotoxicity toward THP-1 cells was determined essentially as already described [26] using the CellTox™ Green Cytotoxicity Assay (Promega), which measures changes in membrane integrity that occur as a result of cell death. THP-1 monocytes grown in colorless RPMI medium (R7509, Sigma) supplemented with L-glutamine (0.03%) were seeded in 96-well plates (1 × 10^5^ cells per well) and differentiated into macrophages. The differentiated macrophages were infected by adding 100 μL of a *B. pertussis* cells in SS medium (containing 5 × 10^6^ cells; MOI 50). After 30 min of co-incubation, non-adherent bacteria were removed by washing, and the remaining extracellular bacteria were killed by incubation in RPMI medium containing 100 μg/mL of polymyxin B sulfate (Sigma) for 30 min. At this time, the infected cells were washed intensely with prewarmed RPMI medium, and the assay was started by adding of CellTox^TM^ reagent according to the manufacturer's protocol. Uninfected cells were treated in the same manner and served as controls. Next, the macrophages were incubated in a Tecan Spark multimode microplate reader (37°C, 5% CO_2_), and the fluorescent signal was measured at Ex/Em = 490 ± 10/520 ± 10 nm for 12 h after addition of the reagent.

### Biofilm formation assay

Biofilm formation was assayed essentially as already described [53]. Briefly, *B. pertussis* cells grown on agar plates were scraped off and suspended in SS medium to a final OD_600_ of 0.3. Triplicate samples of diluted cultures (200 μL) were inoculated in parallel into 96-well non-treated tissue culture plates. The plates were incubated at 37°C for up to three days and at each time point (24, 48, and 72 h), the loosely adherent and planktonic bacteria were discarded by washing the wells vigorously three times with water. Adherent cells were stained with 0.1% solution of crystal violet for 45 min. Plates were washed with water and dried, followed by solubilization of adsorbed crystal violet in 300 μL of 95% ethanol for 15 min. To quantify biofilm formation, 100 μL of stained solution was transferred to a microtitration plate and the *A*_595_ of the crystal violet stain was measured with a multi-well spectrophotometer Epoch (BioTek).

### Complement killing assay

Bacterial cultures were grown overnight to exponential phase (OD_600_ ≈ 1) and diluted in SS medium to 5 × 10^6^ bacteria/mL of culture and supplemented with either intact or heat-inactivated (56°C, 30 min) 10% human serum collected from healthy donors (Sigma #H4522). The cells were incubated in parallel in the presence of both types of sera for 60 min at 37°C in an orbital incubator. Then bactericidal activity was terminated by addition of 10 mM EDTA and serial dilutions of bacterial suspensions were plated onto BG agar and colony forming units (CFU) were counted to assess bacterial survival. Survival was calculated as the percentage of CFU obtained from cultures treated with intact serum compared to CFU from cultures treated with heat-inactivated serum (control, 100% survival).

## Results

### RIL-seq analysis of hfq-mediated RNA-RNA interactions

To test the functionality of FLAG-tagged RNA chaperone Hfq, we co-immunoprecipitated (co-IP) RNAs that associated with Hfq from wt Tohama I cells (*hfq*-WT) and from cells carrying tagged Hfq protein (*hfq*::3xFLAG). Next, we applied quantitative PCR to determine the levels of the small RNA RgtA, whose abundance has been previously shown to be strongly dependent on Hfq [22]. The analysis revealed that while the levels of RgtA in bacterial lysates of wt and tagged cells were comparable, in the co-immunoprecipitated samples the amount of RgtA sRNA in *hfq*::3xFLAG samples was increased more than 20-fold compared to the wt cells (Supplementary Fig. S1). This result proved that the Hfq-dependent sRNA RgtA was enriched in *hfq*::3xFLAG samples and thereby indicated that tagged Hfq is functional.

Next, we performed RIL-seq analysis with pentaplicates of exponentially grown *hfq*-WT cells (negative control) and *hfq*::3xFLAG cells. Immunoprecipitation of Hfq from bacterial lysates, nuclease treatment of Hfq-bound RNA, and ligation of bound RNA molecules was performed following the original RIL-seq protocol [31]. Ligated transcripts recovered from immunoprecipitated Hfq were subjected to Illumina sequencing to identify chimeric fragments.

To gain a general picture of RNA species that associated and co-immunoprecipitated with tagged Hfq, we compared quantity of mapped reads between *hfq*-WT (negative control) and *hfq*::3xFLAG samples by differential expression (DE) analysis. DE analysis revealed that in total 64 Candidate_Transcript RNAs were significantly enriched in *hfq*::3xFLAG samples including sRNA RgtA (Supplementary Table S3). Surprisingly, a large portion of them (44 out of 64) were antisense regulatory RNAs. Among the enriched RNA species we identified also more than 30 tRNAs.

**Figure 1.**
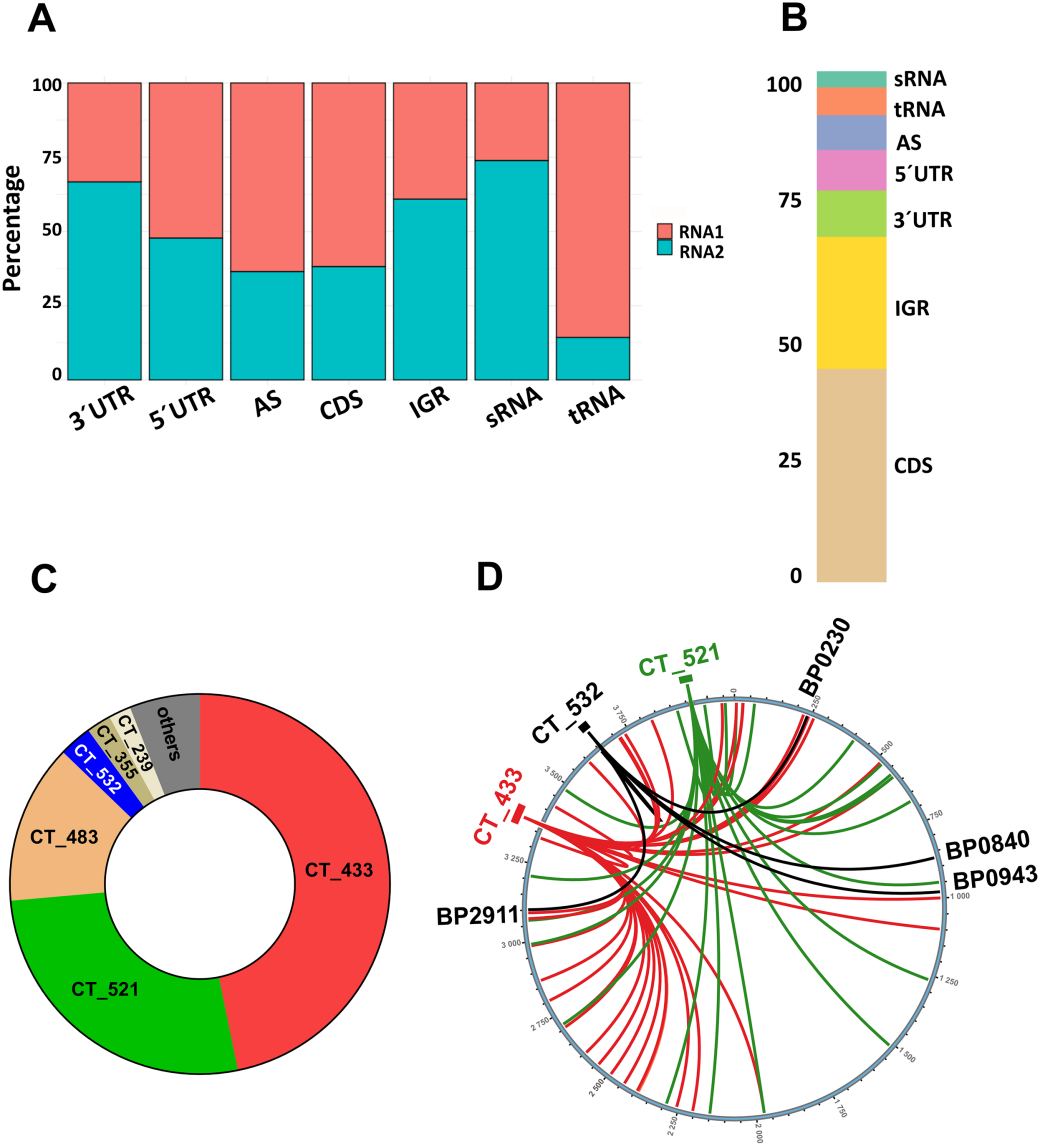
Analysis of the RIL-seq data. (**A**) Distribution of RNA types as first (RNA1) and second (RNA2) RNA in S-chimeric fragments. RNAs of different genomic origin were classified as follows: AS (antisense), 5′UTR, 3′UTR, CDS (coding sequence), and IGR (intergenic region). (**B**) Distribution of RNA types in S-chimeric fragments identified in *hfq*::3xFLAG samples and having sRNA as one of the interaction partners. RNA types are classified as described in panel A. (**C**) The relative abundance of sRNAs detected in S-chimeras determined by dividing the number of chimeric fragments involving a particular RNA by the total number of chimeric fragments containing any sRNA. (**D**) Circos plot of RIL-seq interactions involving CT_532, CT_433, and CT_521 sRNAs. The interactions of CT_532, CT_433 and CT_521 sRNAs are represented by lines connecting the corresponding sRNAs to their targets. Only the targets of CT_532 are shown.

Then we applied computational RIL-seq protocol to map sequenced fragments resulting from ligation of two Hfq-bound RNA molecules (RNA1 and RNA2) to *B. pertussis* genome. In total, five combined *hfq*-WT and five combined *hfq*-3xFLAG samples yielded over 7.7 and 15.7 million Hfq-bound fragments, respectively. In both datasets, approximately 97% of the fragments were classified as “single” fragments, where both RNAs mapped to the same genomic locus, and 3% as “chimeric” fragments, where the RNA pair mates mapped to distinct loci, values that are comparable to other studies [32, 33]. Fisher’s exact test was applied to identify statistically significant chimeric RNA1-RNA2 pairs (S-chimeras) within the RIL-seq dataset (*P* value ≤ 0.05). A total of 68 and 319 S-chimeras were identified in five *hfq*-WT and *hfq*::3xFLAG replicates, respectively (Supplementary Table S4). Among 68 S-chimeras identified in control samples only seven chimeras contained sRNA as one of the interacting partners. Next, we focused on RNA distribution in 319 S-chimeras found in samples with tagged Hfq. Similar to previous RIL-seq analyses, sRNA (in 75% cases) and 3′UTR (in 70% cases) were predominantly found as RNA2 in S-chimeras (Fig. 1A). Next, we analyzed 89 S-chimeras having sRNA as one of the interacting partners (Fig. 1B, Supplementary Table S5). As the most prevalent second interaction partner, we identified coding sequences (42%), followed by intergenic regions (26%), and 5′ and 3′ UTRs of mRNAs (17%).

### Characterization of CT_433 and CT_521 sRNAs

**Figure 2.**
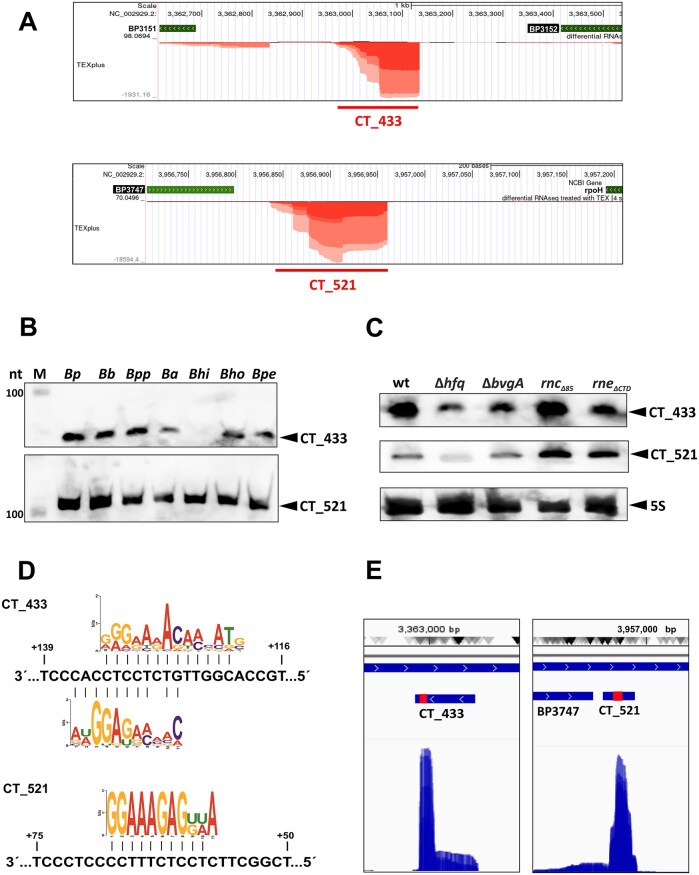
Characterization of the sRNAs CT_433 and CT_521. (**A**) Web browser-derived screenshot of the differential RNA-seq normalized dataset [21] showing the genomic regions between the *bp3151* and *bp3152* genes including CT_433 sRNA (upper panel) and the *bp3747* and *bp3748* (*rpoH*) genes including CT_521 sRNA (lower panel). Graphs display the sequencing depth of the positive and negative strands obtained with the TEX-treated library. The different color intensities represent the different library replicates. The gene annotations are depicted as green arrows. Red bars below indicate the genomic positions of CT_433 and CT_521 sRNAs. (**B**) Conservation of CT_433 (upper panel) and CT_521 (lower panel) sRNAs in different *Bordetella* species. Northern blot analysis was performed using biotinylated probes and total RNA isolated from *B. pertussis* Tohama I (Bp), *B. bronchiseptica* RB50 (Bb), *B. parapertussis* 12 822 (Bpp), *B. avium* CCM 6184 (Ba), *B. hinzii* CCM 2985 (Bhi), *B. holmesii* CCM 4585 (Bho), and *B. petrii* CCM 7166 (Bpe). Biotinylated RNA Century-Plus ladder was loaded as a molecular size marker (M). (**C**) The abundance of CT_433 and CT_521 sRNAs in the wt, Δ*hfq*, Δ*bvgA*, *rnc_Δ85_* and *rne_ΔCTD_* strains. The signals for CT_433 (upper panel), CT_521 (middle panel), and 5S RNA (lower panel, loading control) were detected using biotinylated probes specific for the corresponding RNA. Only the relevant parts of the blots are shown. (**D**) Identification of the common sequence motifs in the putative targets of CT_433 sRNA (upper plot) and CT_521 sRNA (lower plot). Presented is the logo of the motif identified by the MEME suite [47] (shown in 5′ to 3′ direction) and the base pairing with the corresponding sRNA (shown in 3′ to 5′ direction). (**E**) Genome-browser-derived screenshot of all fragment reads mapping to CT_433 (left panel) and CT_521 (right panel) sRNAs. The red rectangles depict the C/T-rich regions that are conserved in all bordetellae. The plot was generated using IGV browser.

Collectively, the analysis revealed that more than 10 different *trans*-encoded sRNAs are part of the S-chimeras, which is a relatively low number compared to previous studies [36, 54, 55]. Surprisingly, though the RgtA sRNA was enriched in samples with tagged Hfq, we did not find it among the S-chimeras with its previously identified target *bp3831* [22]. It is possible that this interaction that was proved *in vitro* is rather weak to be captured by the RIL-seq method *in vivo*. Most chimeric interactions between sRNA and mRNA (> 73%) involved two regulatory RNAs, CT_433 (36 S-chimeric partners) and CT_521 (22 S-chimeric partners) (Fig. 1C, Supplementary Table S5). CT_433 and CT_521 sRNAs were previously identified as approximately 170 nt and 120 nt long transcripts located between the *bp3151* and *bp3152* genes and between the *bp3747* and *rpoH* (*bp3748*) genes, respectively (Fig. 2A). Alignment of the CT_433 and CT_521 sequences revealed that both sRNAs are highly conserved (> 99% sequence identity) in the closely related *B*. *bronchiseptica* and *B. parapertussis*, whereas in other bordetellae and related bacteria only the 3′ region including the terminator is well conserved (Supplementary Figs S2 and S3). Remarkably, these conserved regions upstream of the terminator are extremely C/T-rich. We confirmed this finding by Northern blot analysis using probes derived from the conserved regions. We detected a signal of the expected size for CT_521, whereas we obtained a shorter product with the CT_433-specific probe (Fig. 2B). This is consistent with previous results from Hinton’s group showing that this sRNA is processed by RNase E to a shorter variant [28, 29]. Of note, the abundance of CT_433 in *B. hinziii* is very low. Next, we performed a Northern blot analysis to determine the effects of several important factors such as Hfq, RNase III, RNase E and BvgA on the expression of both sRNAs. This analysis showed that the levels of CT_433 sRNA are reduced in the Δ*hfq*, Δ*bvgA*, and *rne* mutants (Fig. 2C). The abundance of CT_521 is increased in both endonuclease mutants and reduced in the Δ*hfq* mutant (Fig. 2C). These data indicate that both sRNAs are Hfq-dependent, are cleaved by RNase E and the expression of CT_433 appears to be controlled by the *bvg* system. Given the strong conservation and high number of their interactions, we searched for common sequence motifs in the targets of the two sRNAs. The analysis of the RNA1 fragments in the CT_433 and CT_521 sRNA datasets using the MEME algorithm identified a motif that highly resembles Shine-Dalgarno sequence and is essentially complementary to the conserved C/T-rich regions in both sRNAs (Fig. 2D). In addition, most of the CT_433- and CT_521-specific reads mapped to C/T-rich conservative seeds (Fig. 2E).

**Figure 3.**
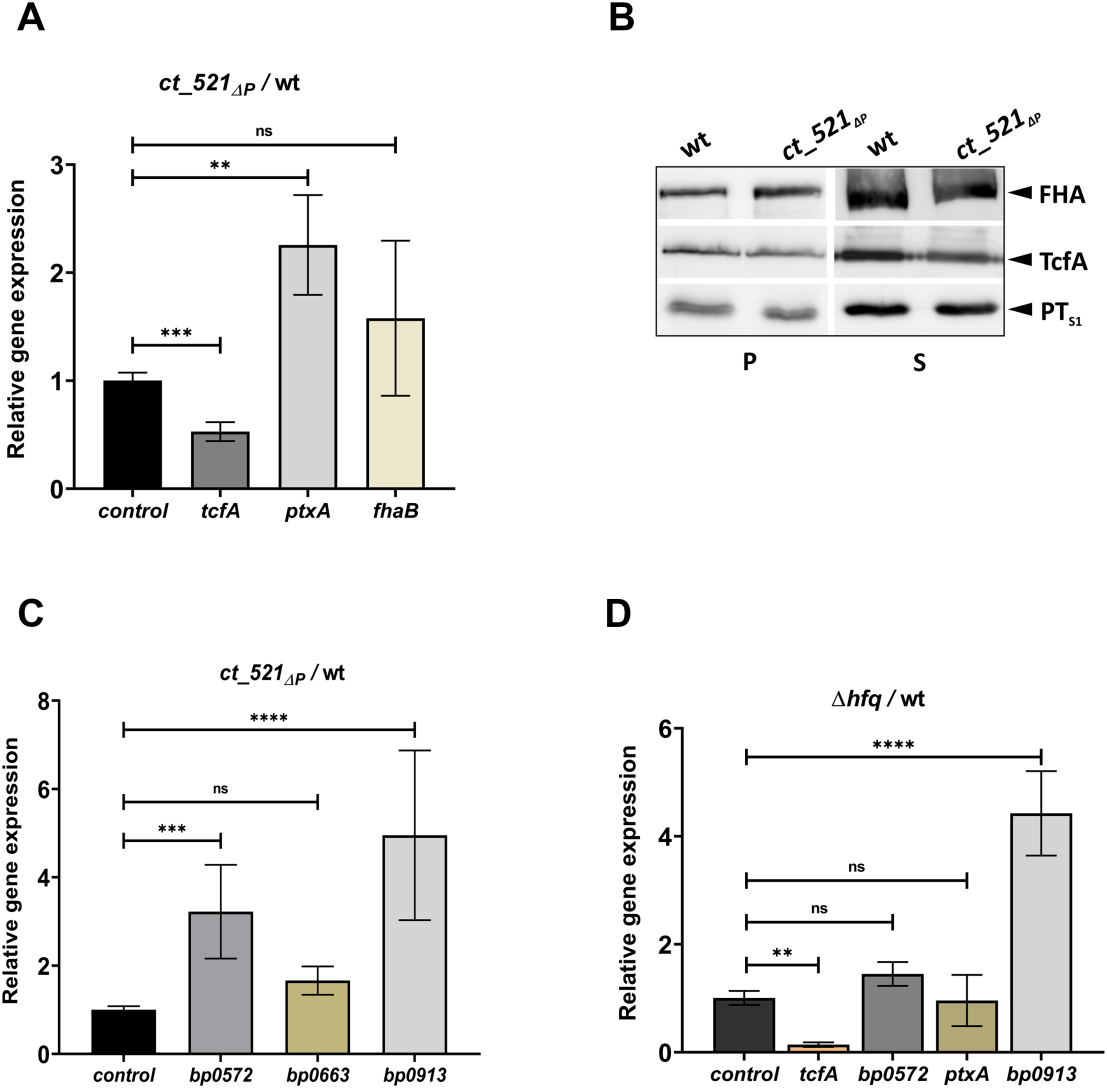
RT-qPCR and Western blot analyses of selected targets of CT_521 sRNA. (**A**) Expression of the virulence genes *tcfA*, *ptxA*, and *fhaB* was compared between the *ct_521_ΔP_* mutant and the wt strain by quantitative PCR. Expression in the wt strain was set to 1. Results are averages of three independent experiments; the error bars represent standard deviations. Statistical analysis was performed using the one-way ANOVA test for multiple comparisons (Dunnett’s test); ns, *P*-value > 0.05; **, *P*-value < 0.005; ***, *P*-value < 0.001. (**B**) Western blot analysis of the production and secretion of TcfA, PTS1, and FHA proteins in the *ct_521_ΔP_* mutant and wt strain. Samples of pelleted cells (P) equivalent to 0.1 OD_600_ unit and precipitated supernatant proteins (S) equivalent to 1 OD_600_ unit were separated on 12.5% SDS-PAGE and analyzed by immunoblotting using antibodies against corresponding proteins. Only the relevant parts of the membrane are shown. (**C**) The expression of *bp0572*, *bp0663*, and *bp0913* genes was compared between the *ct_521_ΔP_* mutant and wt strain by quantitative PCR. The expression in the wt strain was set to 1. The results are averages of three independent experiments; the error bars represent standard deviations. Statistical analysis was performed using the one-way ANOVA test for multiple comparisons (Dunnett’s test); ns, *P*-value > 0.05; ***, *P*-value < 0.0005; ****, *P*-value < 0.0001. (**D**) The expression of *bp0572*, *tcfA*, *ptxA*, and *bp0913* genes was compared between the Δ*hfq* mutant and wt strain by quantitative PCR. The expression in the wt strain was set to 1. The results are averages of three independent experiments; the error bars represent standard deviations. Statistical analysis was performed using the one-way ANOVA test for multiple comparisons (Dunnett’s test); ns, *P*-value > 0.05; **, *P*-value < 0.01; ****, *P*-value < 0.0001.

Among the targets of CT_433, we identified genes encoding autotransporters such as pertactin (*prn*) and Vag8 (*vag8*), the two-component system regulator *ArcA* (*bp0022*) and several enzymes. The CT_521 regulon consists of genes encoding filamentous hemagglutinin (*fhaB*), pertussis toxin subunit S1 (*ptxA*), tracheal colonization factor (*tcfA*), the two-component system regulator PlrR (*bp0572*) and several membrane proteins, among others (Supplementary Table S5). To verify some of the RIL-seq outcomes, we generated the *ct_521_ΔP_* mutant, which lacks the promoter of the sRNA, and tested the expression and production of the three virulence factors *tcfA*, *ptxA* and *fhaB* by RT-qPCR (Fig. 3A) and Western blot (Fig. 3B). These experiments indicate that the expression of the *tcfA* gene and the secretion of the TcfA protein are reduced in the *ct_521_ΔP_* mutant. The expression of the *ptxA* and *fhaB* genes appeared to be increased in the mutant, but the amounts of their gene products did not differ. In addition, we tested by RT-qPCR the expression of three randomly selected targets of CT_521 identified by RIL-seq: *bp0663*, *bp0913* and *bp0572* (Fig. 3C). This analysis indicates that the expression of the response regulator *bp0572* (*plrR*) and the sulfonate transporter *bp0913* is significantly increased in the mutant. Since the abundance of CT_521 was positively affected by Hfq (Fig. 2C), the expression of genes that were significantly affected in the *ct_521_ΔP_* strain was also determined in the Δ*hfq* strain. The expression of the *tcfA* and *bp0913* genes in the Δ*hfq* mutant followed a similar pattern as in the *ct_521_ΔP_*mutant (Fig. 3D).

### Analysis of S-chimeras identifies a novel 3′-UTR-derived sRNA

**Figure 4.**
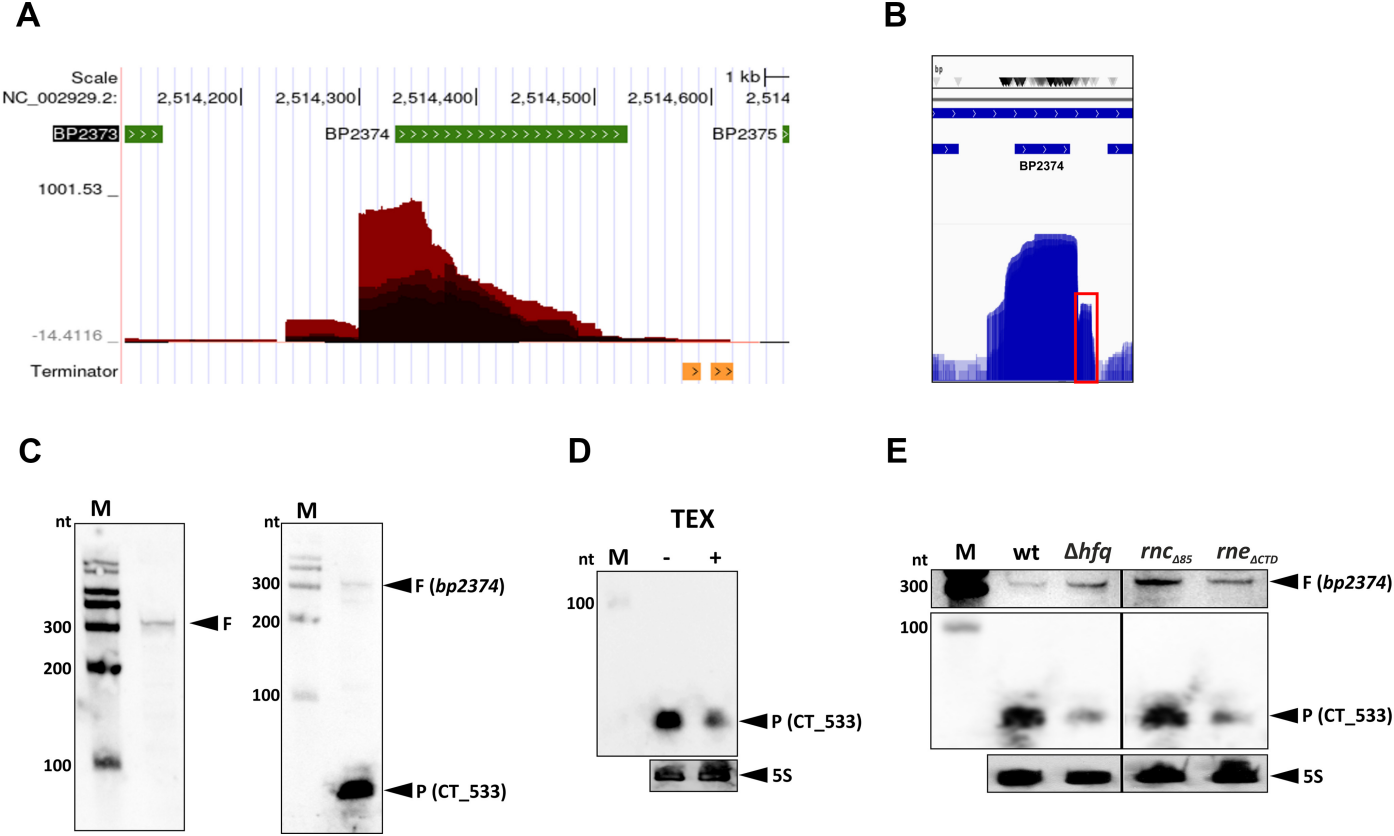
Characterization of the CT_533 and CT_466 transcripts. (**A**) Web browser-derived screenshot of the differential RNA-seq normalized dataset showing the *bp2374* genomic region. The graph displays the sequencing depth of the positive and negative strands obtained with the terminator exonuclease (TEX)-treated library. The different color intensities represent the different library replicates. The gene annotations and terminators are depicted as green and orange arrows, respectively. (**B**) Genome-browser-derived screenshot of all fragment reads mapping to *bp2374* locus. The rectangle depicts the reads mapping to putative sRNA CT_533. The plot was generated using IGV browser. (**C**) Detection of *bp2374*-specific transcripts by Northern blot. Total RNA of *B. pertussis* was probed with biotinylated probes derived from the 5′ (left panel) and the 3′ (right panel) regions of the *bp2374* transcript. Signals corresponding to the full-length transcript (F; *bp2374*) and the processed transcript (P; CT_533) are indicated with arrowheads. Biotinylated RNA Century-Plus ladder was loaded as a molecular size marker (M). (**D**) Equal amounts of untreated (-) and TEX-treated (+) samples of total RNA were probed with a biotinylated probe derived from the 3′ region of the *bp2374* transcript. Signals corresponding to the processed *bp2374* transcript (P, CT_533, upper panel) and 5S RNA (lower panel, loading control) are indicated with arrowheads. Biotinylated RNA Century-Plus ladder was loaded as a molecular size marker (M). (**E**) The abundance of the full-length *bp2374* transcript (F) and the processed transcript (P, CT_533) was assayed by Northern blot. Total RNA isolated from wt, Δ*hfq*, *rnc_Δ85_* and *rne_ΔCTD_* strains was probed with biotinylated probes specific to the 5′ end (upper panel) and to the 3′ end (middle panel) regions of the *bp2374* transcript and 5S RNA (lower panel, loading control). The signals corresponding to full-length and processed transcripts and 5S RNAs are indicated with arrowheads. Biotinylated RNA Century-Plus ladder was loaded as a molecular size marker (M). Only the relevant parts of the blots are shown.

When analyzing the organization of other S-chimeras (*i*.*e*. those that do not contain *bona fide* sRNA), we noticed that the 3′UTR region of the *bp2374* transcript is present as RNA2 in 10 chimeras (Supplementary Table S4). Because 3′UTRs are known genomic reservoirs for sRNA [56, 57], we examined the corresponding region using primary transcriptome data [21]. The data we obtained with the terminator exonuclease (TEX)-treated library, which specifically digests RNA with 5′-monophosphate ends, suggested that this region does not contain an internal promoter (Fig. 4A). Nevertheless, mapping of RIL-seq reads to the *bp2374* locus revealed a small peak in the 3′UTR of the gene (Fig. 4B), suggesting that a putative sRNA might originate from endonuclease processing. Therefore, we performed Northern blot analysis using two probes binding to either the 5′ or 3′ end region of *bp2374*. The 5′end probe identified the full-length *bp2374* transcript of the expected size (≈ 310 nt), whereas the 3′end probe identified only a faint signal of the full-length transcript and a stronger signal of a shorter transcript (Fig. 4C). Moreover, the abundance of the shorter product after treatment with the TEX enzyme was strongly reduced, confirming that this shorter product results from processing (Fig. 4D). Next, we examined the *bp2374*-specific RNA profiles in the wt strain and in the *hfq*, *rne* and *rnc* mutants by Northern blot. These experiments showed that full-length transcript is more abundant in the Δ*hfq*, *rnc_Δ85_* and *rne_ΔCTD_* strains, while the shorter product is less abundant in the Δ*hfq* and *rne_ΔCTD_* mutants and more abundant in the *rnc_Δ85_* mutant (Fig. 4E). These results suggest that the shorter product represents a novel sRNA that is processed from the 3′-UTR region of the *bp2374* transcript by RNase E and whose abundance depends on Hfq. In contrast, steady-state levels of the full-length *bp2374* transcript are increased in the absence of Hfq and RNase III. We have designated the *bp2374*-derived sRNA as CT_533.

We also noted several chimeras consisting of two operonic transcripts bridged by an antisense transcript, *e*.*g*. *bp0465*-*bp0466* and *bp2050*-*bp2051* (Supplementary Fig. S4A) or two overlapping head-to-head oriented transcripts, *e*.*g*. *bp0459*-*bp0460* and *bp0959*-*bp0960*, (Supplementary Fig. S4B). Furthermore, we identified two IS*481*-specific transcripts (Supplementary Fig. S4C), CT_004 and CT_466, present in 10 and 17 S-chimeras, respectively (Supplementary Table S4). The sRNA CT_004 is most likely transcribed from the P_out_ promoter of *bp0023* (IS*481* transposase) and interacts with CDS of several protein-coding genes and two sRNAs. In contrast, CT_466 originates from the intergenic region between the *bp3391* and *bp3392* (IS*481* transposase) genes, but reads into the 3′ portion of the *bp3392* transposase gene (Supplementary Fig. S4C). Thus, the major part of CT_466 is in principle complementary to the transposase mRNA. Indeed, 15 of the 17 partners of CT_466 in S-chimeras are 3′-terminal parts of IS*481*-specific transcripts.

### Characterization of the CT_532 sRNA

**Figure 5.**
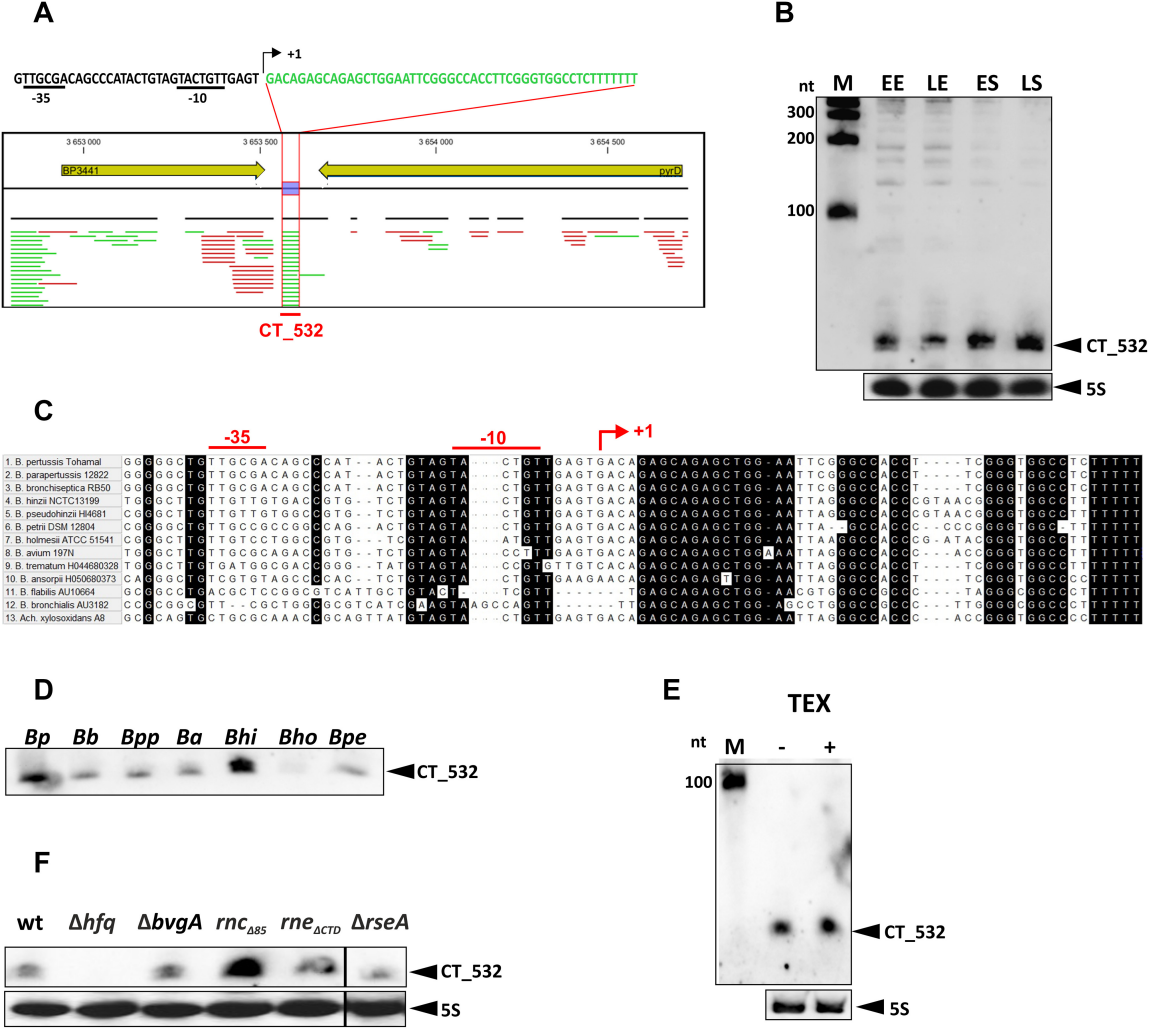
Characterization of the sRNAs CT_532. (**A**) Transcriptomic profiles of the region between the *bp3441* and *pyrD* genes. RNA-seq data [21] were visualized using the CLC Genomics Workbench v10. CT_532-specific reads (in green) are delineated by vertical red lines, the nucleotide sequence map of the ct_532 locus including the *ct_532* sequence (in green), the promoter region (in black) including plausible −35 and −10 sequences (underlined) and the transcription start site of CT_532 (+1; bent arrow) are shown in the plot. (**B**) The accumulation of CT_532 sRNA during different growth phases. Northern blot analysis was performed using total RNA isolated from wt cells grown to early (EE) and late (LE) exponential phase and to early (ES) and late (LS) stationary phase. CT_532-specific signals (upper panel) and 5S RNA-specific signals (lower panel, loading control) were detected using biotinylated probes specific for the corresponding RNA and are indicated with arrowheads. Biotinylated RNA Century-Plus ladder was loaded as a molecular size marker (M). (**C**) *In silico* analysis of CT_532 conservation in different *Bordetella* species and related strains. The DNA sequence corresponding to the *bp3441*-*pyrD* intergenic region of *B. pertussis* Tohama I strain and the genomic sequences of the indicated strains were aligned with the MUSCLE algorithm built in MEGA software. The black background indicates the nucleotides that are highly conserved (> 90% conservation). The promoter region including the plausible −35 and −10 sequences (underlined) and the transcription start site of CT_532 (+1; bent arrow) are shown in red above the plot. (**D**) Conservation of CT_532 sRNAs in different *Bordetella* species. Northern blot analysis was performed using biotinylated probe and total RNA isolated from different *Bordetella* strains described in the legend to Fig. 2B. (**E**) Equal amounts of untreated (−) and terminator exonuclease (TEX)-treated (+) samples of total RNA were analyzed with a CT_532-and 5S RNA-specific biotinylated probes. The signals corresponding to CT_532 and 5S RNAs are indicated with arrowheads. Biotinylated RNA Century-Plus ladder was loaded as a molecular size marker (M). (**F**) The abundance of CT_532 in the wt, Δ*hfq*, Δ*bvgA*, *rnc_Δ85_*, *rne_ΔCTD_*, and Δ*rseA* strains. Signals for CT_532 and 5S RNA (lower panel, loading control) were detected using biotinylated probes specific for the corresponding RNA and are indicated with arrowheads. Only the relevant parts of the blots are shown.

Among the sRNAs with a lower number of interactions we found CT_532 (4 S-chimeric partners). This sRNA was originally predicted by different algorithms and thus named Pred285. In the interest of uniform nomenclature, the Pred285 sRNA was renamed CT_532. CT_532 is located in the intergenic region between *bp3441* and *bp3442* (*pyrD*) and preceded by a plausible and optimally spaced -35 and -10 promoter regions (Fig. 5A). Northern blot analysis revealed that CT_532 can be detected as a double band with an approximate size of 47 nt and its expression is moderately increased in late stationary phase (Fig. 5B). Furthermore, *in silico* (Fig. 5C) and Northern blot (Fig. 5D) data indicate that CT_532 is well conserved in other *Bordetella* species, only in *B. holmesii* the abundance is rather low. Although we identified a plausible promoter for CT_532, the double-banded profile of CT_532 suggested that this sRNA may be processed. Therefore, TEX nuclease-treated and untreated total RNA samples were analyzed with a CT_532-specific probe to assay whether the CT_532 represents a processed sRNA. This assay showed that CT_532 is not degraded by TEX and most likely represents a primary transcript (Fig. 5E).

Next, we determined the amounts of CT_532 in different mutants, as we did for other sRNAs. Northern blot analysis revealed that the levels of CT_532 are strongly decreased in the *hfq* mutant, whereas the levels in the *rnc* mutant are increased (Fig. 5F). These data indicate that CT_532 is an Hfq-dependent sRNA and is likely degraded by RNase III. The expression of MicA in *E. coli* and *Salmonella* is controlled by the product of the *rpoE* gene, the sigma factor E (σ^E^) [58–60]. The *rpoE* mutant of *B. pertussis* is not viable [61] and therefore, to test the possible involvement of the σ^E^ factor in the control of CT_532 expression, we generated the Δ*rseA* mutant, which lacks the anti-σ^E^ factor RseA. Nevertheless, the Δ*rseA* mutant produced similar levels of CT_532 as the wt strain (Fig. 5F).

### CT_532 is homologous to MicA sRNA of *E. coli*

**Figure 6.**
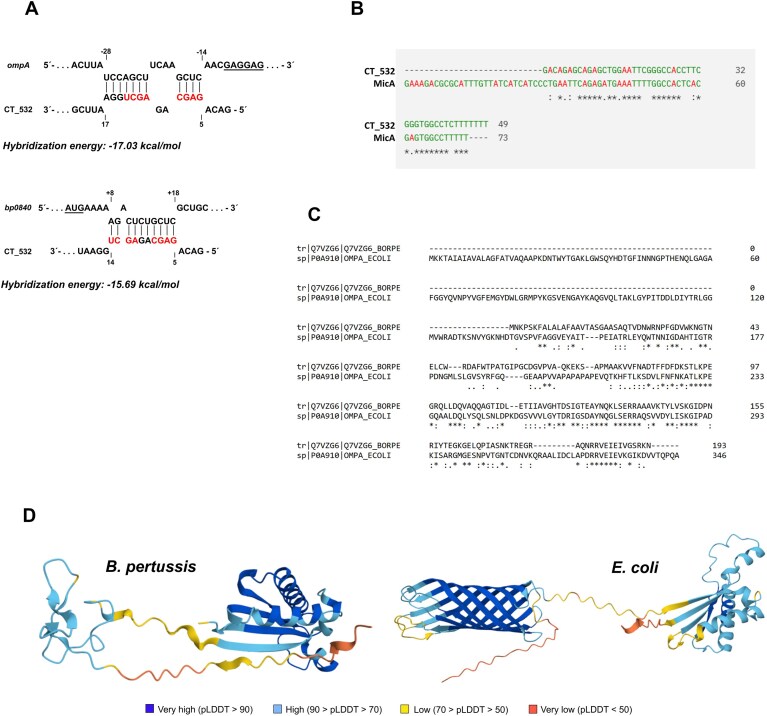
Interactions of CT_532 with its targets and sequence similarity of CT_532 and OmpA with *E. coli* homologs. (**A**) The base-pairing interactions between CT_532 and the 5′UTR of *ompA* (upper panel) and *bp0840* (lower panel) were predicted with IntaRNA program [62]. Numbers above indicate nucleotide position relative the start codon of the mRNA (A being + 1). Numbers below indicate nucleotide position in CT_532. The Shine-Dalgarno sequence of *ompA* mRNA and AUG initiation codon of *bp0840* mRNA are underlined. Nucleotides of CT_532 that participate in both interactions are shown in red. (**B**) Sequence alignment of CT_532 sRNA of *B. pertussis* and MicA of *E. coli* was performed using the Clustal Omega program v. 1.2.4. Nucleotide residues that are identical in both sRNAs are depicted with asterisks. (**C**) Sequence alignment of OmpA porin from *B. pertussis* (CAE41245.1) and OmpA from *E. coli* (NP_415477.1) was performed with the Emboss Needle program (https://www.ebi.ac.uk/jdispatcher/psa/emboss_needle). Amino acid residues that are identical in both proteins are depicted with asterisks. (**D**) Ribbon diagrams of OmpA proteins from *B. pertussis* (left panel, Uniprot code Q7VZG6) and *E. coli* (right panel, Uniprot code P0A910) predicted by AlphaFold including confidence score.

Our RIL-seq analysis suggested that CT_532 interacts with two mRNAs encoding outer membrane proteins (OMPs), namely BP0840, a porin, and BP0943, which is annotated as OmpA in the Uniprot database (accession number Q7VZG6) (Fig. 1D), as well as the pseudogene *bp0230* and gene coding for a putative DNA-binding protein Bph3 (*bp2911*). Therefore, we have extracted the coordinates of CT_532 and *omp* transcripts from S-chimeras and searched for their possible complementarity using IntaRNA, a program for rapid and accurate prediction of interactions between two RNA molecules [62]. This program suggested an interaction between the 5′-region of CT_532 (nt +5 to +14) and a region downstream of the AUG codon of *bp0840* mRNA (nt +8 to +18) and between CT_532 (nt +5 to +17) and a region immediately upstream of the Shine-Dalgarno sequence of *ompA* mRNA (nt −28 to −14) (Fig. 6A). These *in silico* predictions suggested that CT_532 binds in close proximity to the ribosome binding site of the *ompA* transcript and may negatively affect *ompA* translation. The expression and production of outer membrane proteins is repressed in stressed bacteria by various sRNAs such as MicF, MicA or RybB [63]. We determined the sequence homology of CT_532 with known regulators of OMPs and found that CT_532 might represent a shorter variant of MicA, as it shares ≈ 60% identity with the last 46 nt of the *E. coli* sRNA MicA (Fig. 6B). Notably, the 193 amino acid-long OmpA of *B. pertussis* is substantially smaller than the OmpA porin of *E. coli* (346 amino acid residues) and shares 37.8% identity and 53.3% similarity with the *C*-terminal part of the *E. coli* OmpA protein (Fig. 6C). Indeed, the AlphaFold predictions of both proteins clearly show that OmpA of *B. pertussis* lacks the *N*-terminal β-barrel domain, which has been shown to be essential for OmpA function (Fig. 6D) [64].

### CT_532 affects the expression of *ompA* and is induced by temperature shocks

**Figure 7.**
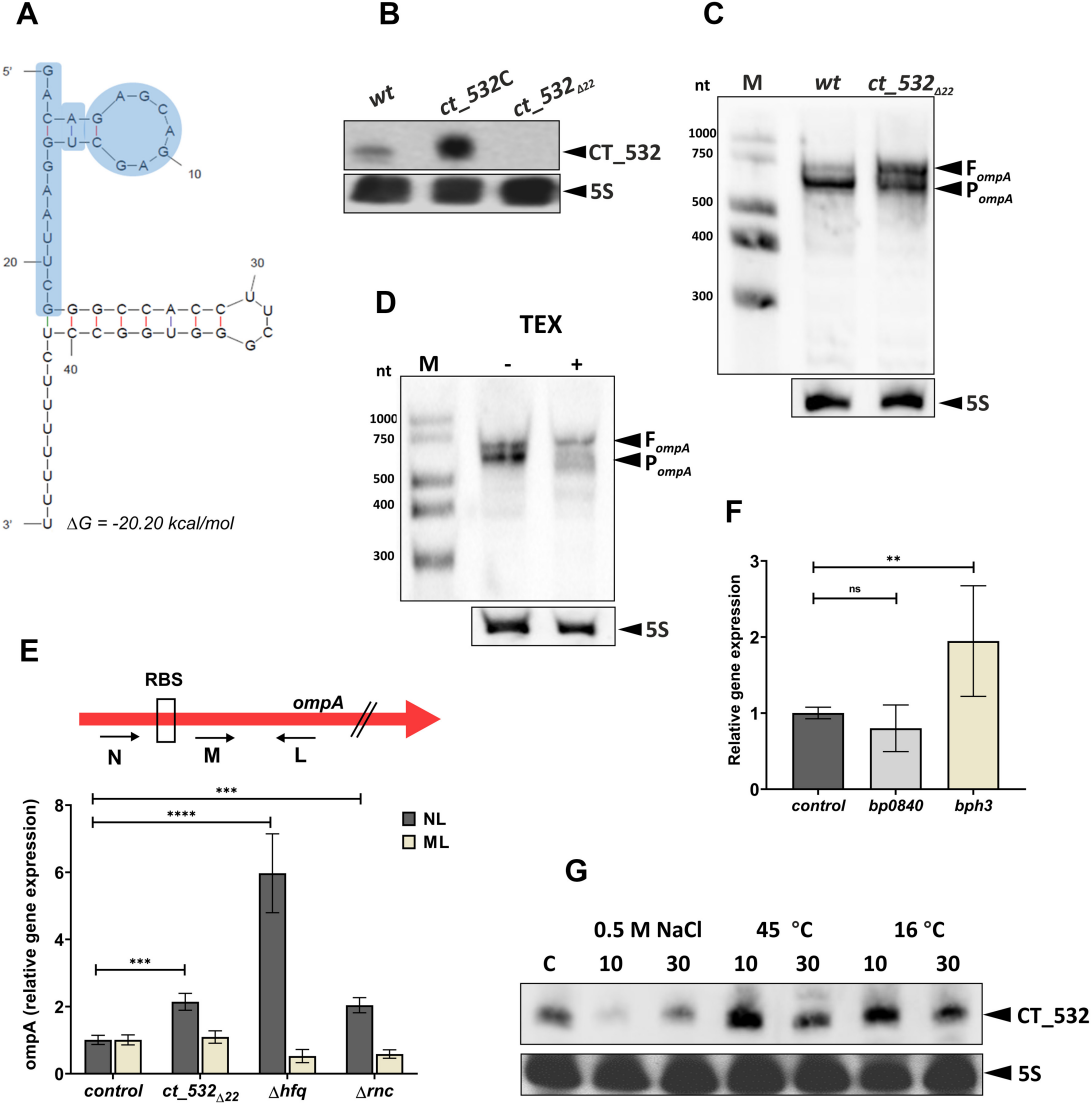
CT_532 affects the expression of *ompA* and is induced by temperature shock (**A**) The predicted secondary structure of CT_532 was generated using the mfold program [90]. The deletion of 22 nucleotides in the *ct_532_Δ22_* mutant is depicted. (**B**) Complementation of the *ct_532*_*Δ*22_ mutant with a plasmid-borne *ct_532* allele results in strain *ct_532C*. Total RNA isolated from wt, *ct_532C*, and *ct_532_Δ_*_22_ strains was probed with biotinylated probes to detect CT_532 (upper panel) and 5S RNA (lower panel) signals. The signals corresponding to CT_532 and 5S RNAs are indicated with arrowheads. (**C**) Identification of full-length (F) and processed (P) *ompA* transcripts in the wt strain and *ct_532_Δ_*_22_ mutant. The samples of total RNA were probed with an *ompA*-specific (upper panel) and 5S RNA-specific (lower panel) biotinylated probes. Signals corresponding to full-length (F) and processed (P) *ompA* transcripts and 5S RNA are indicated with arrowheads. (**D**) Equal amounts of untreated (-) and terminator exonuclease (TEX)-treated (+) samples of total RNA were probed with an *ompA*-specific (upper panel) and 5S RNA-specific (lower panel) biotinylated probes. Signals corresponding to full-length (F) and processed (P) *ompA* transcripts and 5S RNA are indicated with arrowheads. (**E**) Quantification of full-length (F) and processed (P) *ompA* transcripts in the wt strain and *ct_532_Δ_*_22,_ Δ*hfq*, and *rnc_Δ85_* mutants. RT-qPCR was performed with either N and L primers (full-length transcript, NL) or M and L primers (processed transcript, ML). Expression in the wt strain was set to 1. Results are averages of three independent experiments; the error bars represent standard deviations. Statistical analysis was performed using the two-way ANOVA test for multiple comparisons (Sidak's test); ***, *P*-value < 0.001; ****, *P*-value < 0.0001. (**F**) The expression of *bp0840* and *bph3* genes was compared between the *ct_532_Δ_*_22_ mutant and wt strain by quantitative PCR. The expression in the wt strain was set to 1. The results are averages of three independent experiments; the error bars represent standard deviations. Statistical analysis was performed using the one-way ANOVA test for multiple comparisons (Dunnett's test); ns, *P*-value > 0.05; **, *P*-value < 0.01. (**G**) *B. pertussis* cells were grown at 37°C to mid exponential phase and then treated with 0.5 M NaCl or by transfer to 16°C or 45°C to induce osmotic, cold or heat shock. Total RNA isolated from cells 10 and 30 min after shock was used to detect CT_532 (upper panel) and 5S RNAs (lower panel). Untreated cells grown at 37°C were used as control (C).

To investigate the possible role of CT_532 in the expression of *omp* genes, we constructed strain *ct_532_Δ_*_22_, which lacks the first 22 nt of sRNA (Fig. 7A). This deletion was designed based on the results of RIL-seq data and IntaRNA analyzes, indicating that this region interacts with *omp* targets. In addition, the promoter and terminator sequences were left intact to avoid a polar effect on downstream genes. The deletion was complemented in the *ct_532*C strain, which carries a plasmid-borne copy of the *ct_532* gene including its plausible promoter and terminator. The complemented mutant produced increased amounts of sRNA compared to the wt strain (Fig. 7B). Together with the result of TEX treatment of CT_532 (Fig. 5E), this experiment confirms that CT_532 is not derived from processing and is transcribed from its own promoter.

To validate the functional relevance of the *ompA*-CT_532 chimera, we analyzed *ompA* transcript levels in the wt strain and in the *ct_532_Δ2_*_2_ mutant by Northern blot. The *ompA*-specific signals could be detected as two bands of the expected size (> 700 nt), suggesting that *the ompA* transcript is processed (Fig. 7C). Interestingly, the full-length product was more abundant in the *ct_532_Δ2_*_2_ mutant. Treatment with TEX nuclease showed that the lower, possibly processed transcript is degraded by TEX (Fig. 7D), while the amount of the full-length transcript remained intact. Since Hfq and RNase III affected the amounts of CT_532 (Fig. 5F), we quantified the full-length and processed forms of the *ompA* transcript in the wt, *ct_532_Δ2_*_2_, Δ*hfq* and *rnc_Δ85_* strains. Thus, we performed RT-qPCR analyses with two different forward primers binding to either the region upstream of the putative interaction with CT_532 (N primer) or downstream (M primer) (Fig. 7E). This analysis revealed that the amount of the longer PCR product is significantly increased in the *ct_532_Δ2_*_2_, *rnc_Δ85_* and especially in the Δ*hfq* mutant compared to the wt strain (Fig. 7E). In contrast, the quantity of the shorter PCR product did not change significantly in any strain. These data suggest that the CT_532/*ompA* duplex is cleaved by RNase III and Hfq attenuates the levels of *ompA* transcript. We hypothesize that both activities negatively affect the production of the OmpA protein. In addition, we tested the expression of additional targets of CT_532 identified by RIL-seq, *bp0840* and *bph3* genes. The expression of *bp0840* did not change significantly, but the expression of the *bph3* gene was moderately increased in the *ct_532_Δ22_* mutant (Fig. 7F). To prove that CT_532-specific attenuation translated into altered amounts of OmpA protein, we performed a label-free proteomic analysis with wt and *ct_532_Δ2_*_2_ strains. This analysis revealed only a few proteins whose quantity was changed in the mutant, but none of the changes were statistically significant (Supplementary Table S6). Nevertheless, the abundance of the OmpA protein in the *ct_532*_Δ22_ mutant was increased by 30% compared to the wt strain.

MicA expression is known to be modulated by various membrane stressors such as antimicrobial peptides, heat shock, osmotic, pH and ethanol [59, 60]. Therefore, we tested the effect of stressors on the expression of CT_532. Bacterial cells were grown to mid-exponential phase and exposed to stress for 10 and 30 min. Total RNA isolated from the stressed cells and the untreated control sample was analyzed by Northern blot. While polymyxin B and ethanol stress did not affect CT_532 levels (Supplementary Fig. S5A), 0.5 M NaCl strongly reduced the production of CT_532 and both heat shock and cold shock resulted in moderately increased levels of CT_532 (Fig. 7G). In general, the modulatory effects observed 10 min after the introduction of stress conditions were stronger than those observed after 30 min, suggesting that the expression is affected by the stressors only transiently.

### Phenotypic characterization of the *ct_532_Δ22_* mutant

MicA function is linked to outer membrane integrity under stress conditions and our data indicated that CT_532 expression can be induced by suboptimal growth temperature. Therefore, we tested the growth properties and viability of the wt strain and the *ct_532_Δ22_* mutant upon cold and heat shocks, but these experiments did not reveal a significant and reproducible phenotype associated with the deletion of 22 nt in the *ct_532* locus (Supplementary Fig. S5).

**Figure 8.**
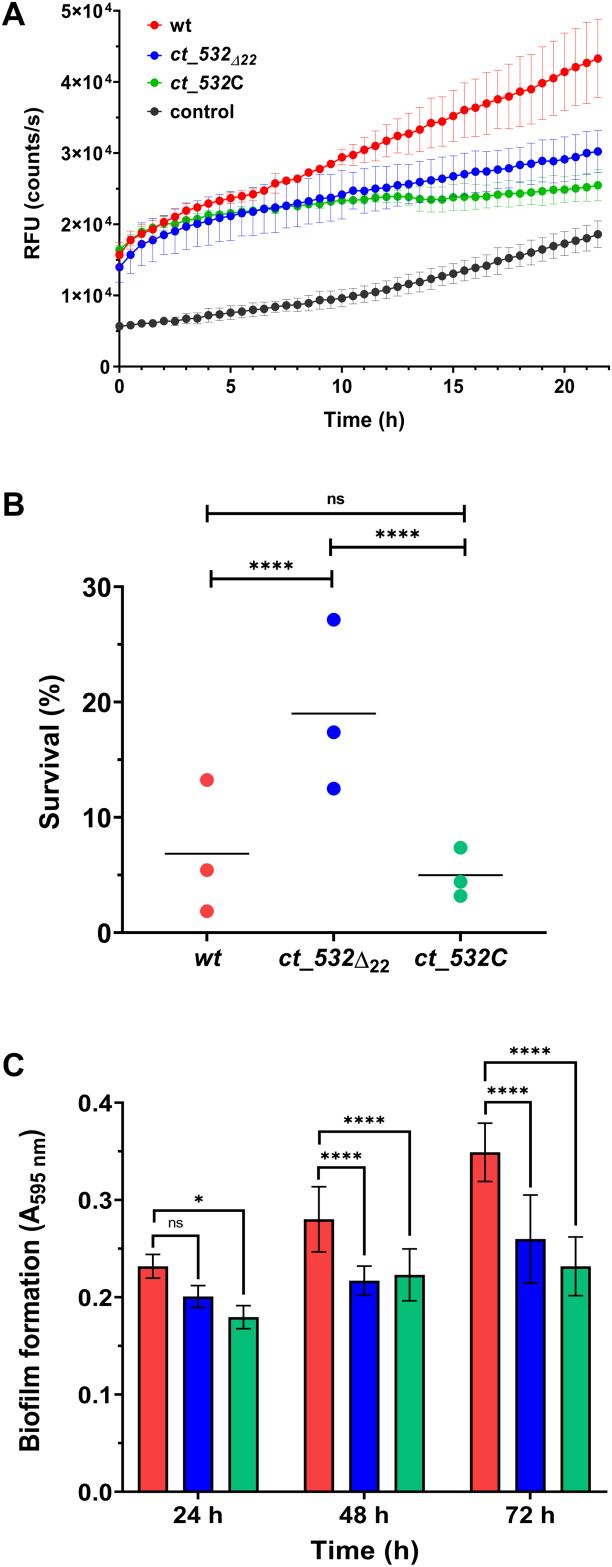
The impact of CT_532 sRNA on cytotoxicity, resistance to complement and biofilm formation. (**A**) Cytotoxicity of the wt strain (red), the *ct_532*_*Δ*22_ mutant (blue) and the complemented mutant (*ct_532*C strain, green) toward THP-1 macrophages. Differentiated macrophages were infected in biological triplicates with *B. pertussis* strains with an MOI of 50, uninfected cells (black) served as control. Immediately after the addition of the fluorescent dye, THP-1 cells were incubated for 22 h (37°C, 5% CO_2_) in the microplate reader. During incubation, the fluorescence of the samples, which is proportional to cytotoxicity, was measured every 30 min. The graph shows the mean values and the standard errors of the means. The result is representative for three independent experiments. (**B**) Resistance to complement of the wt strain (red), the *ct_532*_*Δ*22_ mutant (blue) and the complemented mutant(green). Bacterial cells (10^6^/mL) were incubated in parallel in 10% untreated and heat-inactivated human serum for 60 min at 37°C. Serial dilutions of the cultures were plated to count viable cells. The CFU in cultures incubated with heat-inactivated serum was set to 100%. The plot shows data from three independent experiments performed in triplicate. Statistical analysis was performed using the two-way ANOVA test for multiple comparisons (Tukey’s test); ns, *P*-value > 0.05; ****, *P*-value < 0.0001. (**C**) Biofilm formation was tested in tetraplicates of the wt (red), *ct_532*_*Δ*22_ mutant (blue) and complemented *ct_532*C mutant (green) over three days under static aerobic conditions at 37°C. Biofilm formation was determined as *A*_595_ of the crystal violet staining. The results are averages of three independent experiments; the error bars represent standard deviations. Statistical analysis was performed using the two-way ANOVA test for multiple comparisons (Dunnett’s test); ns, *P*-value > 0.05; *, *P*-value < 0.02; ****, *P*-value < 0.0001.

During infection, bacterial pathogens are exposed to combined stress conditions resulting from a harsh environment within host cells. Low pH, reactive oxygen species and antimicrobial peptides exert strong effects on membrane integrity and thereby impair bacterial survival. Thus, we tested whether CT_532 benefits *B. pertussis* cells during infection of human immune cells. We infected human monocyte-derived macrophages THP-1 with the wt strain and the *ct_532_Δ22_* and *ct_532*C mutants at multiplicity of infection 50 (50 bacteria per macrophage) and examined the capacity of the pathogen cells to induce cytotoxic effects during infection. Infected macrophages and non-infected control macrophages were incubated for more than 20 h and the fluorescence generated by the binding of the dye to DNA in cells with impaired membrane integrity was monitored. The cytotoxic effect of the *ct_532_Δ22_* mutant toward THP-1 cells was greatly reduced compared to the wt strain (Fig. 8A). Interestingly, the cytotoxicity induced by the complemented mutant was even lower than that of the deletion mutant, suggesting that overexpression of CT_532 is also detrimental to pathogenicity of intracellular *B. pertussis* cells.

Since OmpA has been shown to contribute to complement resistance in *E. coli* [65] and our data suggested that CT_532 has a negative effect on *ompA* expression and partially also on OmpA production, we tested the resistance to complement of the wt strain and the *ct_532_Δ22_* and *ct_532*C mutants. All strains were incubated in 10% active and inactivated (control) human serum for 1 h at 37°C and survival rates were determined by plating and counting viable cells. In agreement with published data, the survival rate of the *ct_532_Δ_*_22_ mutant in active serum was significantly higher than that of the wt and complemented mutant (Fig. 8B).

MicA has been shown to be involved in biofilm formation of *Salmonella* [66] and we wondered whether CT_532 would play a similar role in *B. pertussis*. The wt strain and the *ct_532*_*Δ*22_ and *ct_532*C mutants were incubated in SS medium in 96-well plates without shaking and biofilm formation was monitored for 3 days. While all strains produced comparable amounts of biofilm after one day of incubation, the mutants produced significantly less biofilm than the wt strain at later time points (Fig. 8C). Also, compared to both mutants, the wt strain displayed different dynamics in biofilm formation as the *A_595_* values, which correlate with biofilm production, steadily increased over the three days of monitoring. Similar to the cytotoxicity assay, the defect observed in the complemented mutant appeared to be more pronounced after three days of incubation than in the deletion mutant.

## Discussion

In this work, we have analyzed the Hfq-dependent RNA transactions in *B. pertussis* using the RIL-seq method. Hfq plays a significant role in the virulence of *B. pertussis* and therefore we expected that our study could reveal riboregulatory mechanisms involved in the pathogenesis of *B. pertussis*. Compared to studies with similar sequencing depth performed with Gram-negative bacteria [33, 34, 36, 38, 54], our project revealed a lower number of sRNAs involved in significant interactions. This may suggest that the involvement of Hfq in transactions mediated by *trans*-encoded sRNAs in *B. pertussis* is less extensive than in other bacteria. Our analysis of the primary transcriptome revealed that out of 531 Candidate_Transcript RNAs, only 150 were found in intergenic regions [21] and only 40 transcripts exhibited significantly affected abundance in the absence of Hfq [20]. Indeed, most regulatory RNAs in *B. pertussis* are *cis*-encoded, *i.e*. they are expressed antisense to the coding regions. For example, we have previously identified several asRNAs that are transcribed antisense to genes encoding important virulence factors such as the adenylate cyclase toxin and filamentous hemagglutinin [21]. Recently, we have shown that the asRNA Rfi2, which is transcribed antisense to the fimbrial gene *fim2*, reduces Fim2 production and attenuates cytotoxicity toward human macrophages [67]. Our current study revealed that asRNAs are highly enriched in the samples co-immunoprecipitated with the tagged Hfq. This may be surprising as Hfq is thought to preferentially interact with *trans*-encoded sRNAs. However, a previous study focusing on the identification of Hfq-binding motifs using genomic SELEX suggested a role for Hfq in regulation by *cis*-encoded asRNAs [68], and in a recent RIP-seq analysis of Hfq-bound regulatory RNAs of *Clostridioides difficile*, *cis*-asRNAs were among the most abundant classes of regulatory RNAs [69]. However, only seven asRNA-mediated interactions were found among the 89 S-chimeras, suggesting that Hfq is not a critical factor for interactions of asRNAs with their targets.

In this study, we focused on *trans*-encoded sRNAs and we show that more than 73% of the significant chimeric interactions involve two *trans*-encoded sRNAs, CT_433 and CT_521. These two sRNAs share several features, such as strong conservation in bordetellae, increased abundance in the presence of Hfq, a C/T-rich seed domain upstream of the terminator, and an A/G-rich common target mRNA motif resembling the ribosome binding site. This may explain the large number of the target genes and suggests that CT_433 and CT_521 represent a central riboregulatory hubs of *B. pertussis*. Therefore, it will be important to determine how their expression is controlled under different stress conditions. Nevertheless, previous dual RNA-seq analysis showed that none of these sRNAs is induced in internalized bacteria during infection of human macrophages [26]. During the revision of this manuscript, Sim *et al.* published a preprint focusing on the characterization of CT_433 (referred to as S17 sRNA in the preprint) [29]. Interestingly, four CT_433 targets identified by our RIL-seq analysis, *bp2271*, *bp2642A*, *bp2811*, and *bp3501*, are among the 11 highly conserved targets of S17 sRNA identified by RNA-seq analysis [29]. As for the putative targets of CT_521, we identified virulence factors including those present in current acellular vaccines, such as TcfA, FHA and PT. Our data suggest that CT_521 could be required for the expression and secretion of the TcfA. Therefore, it will be of utmost importance to investigate the role of CT_433 and CT_521 sRNAs in the fitness and virulence of *B. pertussis*.

Importantly, RIL-seq analysis revealed a novel sRNA CT_533 that is apparently derived from the 3′end of the *bp2374* transcript. This finding illustrates the previously described capacity of RIL-seq to identify novel sRNAs [70]. We suggest that this sRNA is produced by RNase E processing and we plan to test its involvement in the control of identified target genes in our future experiments. Our data also identified an interesting transcript CT_466 that shares the properties of both sRNA and asRNA as it is transcribed from an intergenic region and reads into a 3′-terminal coding sequence of an IS*481* transposase gene. This “hybrid” regulatory RNA was found in 15 S-chimeras interacting with 3′-terminal CDSs of IS*481* transcripts. To minimize the negative effects on the fitness of bacteria, the transposase expression must be controlled. Similar to the IS*10* element [71], the expression of the IS*481* transposase gene driven by the P_in_ promoter is attenuated by pairing with an asRNA transcribed from the P_out_ promoter. We hypothesize that CT_466 base pairs with the 3′-terminal part of the transposase transcript and thereby also contributes to the control of transposase expression. Interestingly, Hfq has been shown to facilitate the base pairing between P_in_ and P_out_-driven IS*10* transcripts [72] and our study revealed that CT_466 is one of the most enriched RNAs in *hfq*-3xFLAG samples (Supplementary Table S3).

Intriguingly, in 16 S-chimeras we found tRNA as one of the interacting partners, predominantly as RNA1 (13 S-chimeras). We do not know whether this phenomenon is an artifact, yet more than 30 tRNAs were significantly enriched in *hfq*-3xFLAG samples (Supplementary Table S3). Interestingly, tRNA precursors were shown to co-immunoprecipitate with Hfq [73] and later Hfq was shown to bind tRNAs with high specificity and affinity and contribute to translational fidelity [74]. Of note, the tRNA-derived small RNAs have recently been described as aptamer-like regulatory RNAs in all kingdoms of life [75].

Finally, our analysis revealed interesting interactions between the CT_532 sRNA and two transcripts encoding outer membrane proteins that resemble the interaction of MicA sRNA with *omp* targets in *E. coli*. The interaction of MicA with *ompA* mRNA belongs to one of the first riboregulatory mechanisms to be studied at the molecular level [76, 77]. Our *in silico* analysis showed that CT_532 is highly conserved in bordetellae, shares around 60% similarity with MicA and its expression is transiently induced by cold and heat shocks, whereas osmotic stress has a negative effect. The accumulation of CT_532 is strongly dependent on Hfq, but in contrast to MicA, the expression of CT_532 appears to be independent of RpoE. It should be noted that the MicA sequence, which interacts with the *ompA* mRNA is absent in CT_532 [76, 77], yet our data suggest that a similar interaction, involving a different interaction region, has evolved in *B. pertussis* and possibly in other bordetellae. Intriguingly, the OmpA of *B. pertussis* lacks the *N*-terminal β-barrel, which has recently been shown to be important for envelope’s strength [64]. However, it still possesses the *C*-terminal domain, which interacts non-covalently with peptidoglycan [78] and contributes to resistance to antibiotics [79]. Interestingly, inactivation of the outer membrane porin OprD in *Pseudomonas aeruginosa* led to carbapenem resistance [80]. It remains to be investigated whether loss of the β-barrel of OmpA confers an advantage to *B. pertussis*, but the surface-exposed loops of the β-barrel can serve as a target of the immune system and moreover, OmpA can induce protective immunity against *Shigella flexneri* and *E. coli* in a mouse model of infection [81, 82]. Thus, loss of the *N*-terminal β-barrel of OmpA can be seen as a mechanism that contributes to immune evasion.

Our data suggest that the CT_532 sRNA base pairs with the region near the ribosome binding site of the *ompA* transcript and that this duplex is cleaved by RNase III. The abundance of the *ompA* transcript appears to be strongly reduced by Hfq, possibly beyond its role in CT_532 accumulation, as has also been shown for *E. coli* [83, 84]. We hypothesize that the role of CT_532, similar to MicA, is to reduce the production of OmpA under stress conditions. Our proteomic data support this idea, although the effect is not statistically significant. This could be partly due to the relatively low expression of CT_532 compared to highly expressed *omp* genes, which are among the 30 most highly expressed genes in *B. pertussis* [20]. The role of CT_532 in regulation of *bp0840* expression is less clear as our experiments did not reveal any significant effect. MicA is conserved among the *Enterobacteriaceae* and therefore its function has been hypothesized to be related to the gut environment [85]. In support, a functional analog of MicA, VrrA, was found in *Vibrio cholerae* and was shown to act on *ompA*, as the *vrrA* mutant overproduced the OmpA porin [86]. Based on our data, we hypothesize that *B. pertussis*, a human respiratory pathogen, expresses a functional homolog of MicA, CT_532 sRNA, that interacts with *ompA* and responds to various membrane stressors. Thus, it appears that sRNA-mediated control of outer membrane porins is not restricted to a specific niche in the human host.

Mutants lacking sRNA genes are often notorious for lacking distinct phenotypes under experimental *in vitro* conditions [87], and this is also partially true for MicA. Although induction of MicA expression in the presence of various stressors and MicA interaction with the *ompA* transcript have been intensively studied and thoroughly characterized, none of the *micA* mutants showed a significant growth deffects, reduced fitness, or impaired survival in the presence of membrane stressors [76, 77]. Similarly, we did not detect a significant growth phenotype in the *ct_532* mutant in the presence of stressors that affected CT_532 expression, most likely due to only transient overexpression during stress.

In addition, it is often difficult to directly link the phenotypic traits of *micA* mutants, such as altered biofilm formation, motility and virulence, to known MicA targets. For example, overexpression of MicA from a plasmid increased motility of *E. coli* by 50%, but the mechanism is unknown as flagella regulators were not affected by MicA [88]. MicA was implicated in biofilm formation of *Salmonella*, as overexpression and to a lesser extent depletion of MicA resulted in reduced biofilm formation [66]. In other words, both lack of expression and overexpression impaired biofilm formation. This phenotype led to the assumption that levels of MicA must be balanced for proper biofilm formation. Our results are highly consistent with these results, as both the *ct_532_Δ22_* mutant and its complemented variant, which overproduces CT_532, exhibited significantly reduced biofilm levels. Our results also indicate that the absence of first 22 nt of CT_532 leads to reduced cytotoxicity of internalized *B. pertussis* cells. Our recent dual RNA-seq analysis revealed that the expression of porin genes such as *bp0840*, *ompW*, and *ompA* was decreased in intracellular *B. pertussis* cells during infection of human macrophages [26]. We understood this observation as an adaptation of the pathogen cells leading to reduced transport of small molecules such as antimicrobial peptides. Thus, the reduced cytotoxicity of the *ct_532_Δ22_* mutant likely results from an increased expression of the *ompA* gene. Similar to biofilm formation, overproduction of CT_532 resulted in even further reduced cytotoxicity, suggesting that overexpression of CT_532 could be as detrimental to virulence as its deletion. In contrast, the *ct_532*_*Δ*22_ mutant exhibits higher resistance to complement killing compared to the wt strain or the complemented mutant, indicating the importance of OmpA for membrane stability. Evidently, partial deletion of CT_532 can be both beneficial and detrimental for *B. pertussis* cells. We hypothesize that the phenotypic outcome of the CT_532-*ompA* interaction is largely dependent on the extent and duration of sRNA expression and the nature of the stress environment.

## Supplementary Material

gkaf614_Supplemental_Files

## Data Availability

RNA-seq data used for RIL-seq and DE-seq analyses are available at the European Nucleotide Archive under project accession number PRJEB79241. The mass spectrometry proteomics data have been deposited to the ProteomeXchange Consortium via the PRIDE [89] partner repository with the dataset identifier PXD063711.
