# Supplementary material for: Global analysis of the Hfq-mediated RNA interactome discovers a MicA homolog that affects the cytotoxicity, biofilm formation, and resistance to complement of Bordetella pertussis
Source: Nucleic Acids Res. 2025 Jul 8;53(13):gkaf614. doi: 10.1093/nar/gkaf614 (PMC12235514; doi:10.1093/nar/gkaf614)
Supplement: gkaf614_Supplemental_Files [file gkaf614_supplemental_files.zip › Kumar et al Supplementary files.pdf]

Supplementary Materials for

Global analysis of the Hfq-mediated RNA interactome discovers a MicA homolog that affects the cytotoxicity, biofilm formation and resistance to complement of *Bordetella pertussis*

Dilip Kumar[1#], Martin Beles[1#], Argha Saha[1#], Ilona Procházková[1], Ana Dienstbier[1], Jakub Držmíšek[1], Jan Čapek[1], Ivana Čurnová[1], David Hot[2], Denisa Petráčková[1*], and Branislav Večerek[1*]

This PDF includes:

Supplementary Tables S1, S2

Supplementary Figures S1-S5

Supplementary Table 1. Strains and plasmids used in the study

Strain	Description	Reference
Escherichia coli		
SM10(λ*pir*)	Maintenance and mobilization of pSS4245 vector	[1]
XL1 blue	Cloning strain	Promega
Bordetella pertussis		
Tohama I	Wild-type strain	[2]
ct_532$_{\Delta 22}$	Tohama I strain carrying 22-nt deletion of the *ct_532* gene	This study
*ct_532*C	*ct_532*$_{\Delta 22}$ strain carrying pBBRPred285-borne *ct_532* allele	This study
ct_521$_{\Delta P}$	Tohama I strain carrying 12-nt deletion in the promoter region of the *ct_521* allele	This study
rnc$_{\Delta 85}$	Tohama I strain carrying in-frame deletion corresponding to aa residues 2-85 of RNase III	This study
rne$_{\Delta CTD}$	Tohama I strain carrying in-frame deletion corresponding to the C-terminal domain of RNase E	This study
Δ*rseA*	Tohama I strain carrying in-frame deletion of the *rseA* gene	This study
Δ*hfq*	Tohama I strain carrying in-frame deletion of the *hfq* gene	[3]
Δ*bvgA*	Tohama I strain carrying in-frame deletion of the *bvgA* gene	[4]
Bordetella bronchiseptica RB50	Rabbit isolate	[5]
Bordetella parapertussis 12822	Ovine isolate	[6]
Bordetella avium CCM 6184		CCM*
Bordetella hinzii CCM 2985		CCM
Bordetella holmesii CCM 4585		CCM
Bordetella petrii CCM 7166		CCM
**Plasmid**	**Description**	**Reference**
pSS4245	Conjugation vector for allelic exchange in *Bordetella pertussis*	[7]
pBBRMCS1	Cloning vector that replicates in *Bordetella pertussis*	[8]
pBBRCT_532	pBBRMCS1 carrying *ct_532* allele	This study

***** Czechoslovak Collection of Microorganisms (https://ccm.sci.muni.cz/en)

1. Simon, R., U. Priefer, and A. Pühler, *A Broad Host Range Mobilization System for In Vivo Genetic Engineering: Transposon Mutagenesis in Gram Negative Bacteria.* Biotechnology, 1983. **1** p. 784–791.
2. Kasuga, T., et al., *Studies on Haemophilis pertussis. III. Some properties of each phase of H. pertussis.* Kitasato Arch Exp Med, 1954. **27**(3): p. 37-47.

3. Bibova, I., et al., *The RNA chaperone Hfq is required for virulence of Bordetella pertussis.* Infect Immun, 2013. **81**(11): p. 4081-90.
4. Keidel, K., et al., *Signal transduction-dependent small regulatory RNA is involved in glutamate metabolism of the human pathogen Bordetella pertussis.* RNA, 2018. **24**(11): p. 1530-1541.
5. Cotter, P.A. and J.F. Miller, *BvgAS-mediated signal transduction: analysis of phase-locked regulatory mutants of Bordetella bronchiseptica in a rabbit model.* Infect Immun, 1994. **62**(8): p. 3381-90.
6. Heininger, U., et al., *Comparative phenotypic analysis of the Bordetella parapertussis isolate chosen for genomic sequencing.* Infect Immun, 2002. **70**(7): p. 3777-84.
7. Inatsuka, C.S., et al., *Pertactin is required for Bordetella species to resist neutrophil-mediated clearance.* Infect Immun, 2010. **78**(7): p. 2901-9.
8. Kovach, M.E., et al., *pBBR1MCS: a broad-host-range cloning vector.* Biotechniques, 1994. **16**(5): p. 800-2.

Supplementary Table 2. Primers and probes used in this study

Purpose of the primers or probes	Sequence (5′-> 3′)*
Construction of the *ct_532*Δ_{22} mutant (upstream region)	F: T*GAATTC*TTAGGTCCGTAGAATGGCAAT<br>R: T*GCTAGC*ACTCAACAGTACTACAGTATGGGCT
Construction of the *ct_532*Δ_{22} mutant (downstream region)	F: A*GCTAGC*CACCTTCGGGTGGCCTC<br>R: T*GAATTC*AATGTGCAGCGCAGCCAGT
Construction of the plasmid-borne *ct_532* allele	F: GTCGCGACCAAGGCCG<br>R: GCTGAGGCGGGATCCC
Construction of the *ct_521*Δ_{P} mutant (upstream region)	F: GTGTCACTAGTCGAATTCATCATCCGCAACTGGC<br>R: TTATAGCTAGCCACGAGCCGCGATTTAGTTTCCA
Construction of the *ct_521*Δ_{P} mutant (downstream region)	F: ATATAGCTAGCCGTAGACCGCGAACTTGGCC<br>R: AATTAACTAGTACGCGCTGCTGCTCACCG
Construction of the *rnc*Δ_{85} mutant (upstream region)	F: TAT*GAATTC*GAATACTGGAAGCAGATCGGCG<br>R: ATA*GCTAGC*TGACGCACAAAGCGGGTTGCGGCAG
Construction of the *rnc*Δ_{85} mutant (downstream region)	F: ATA*GCTAGC*TTCGGTCTTGGCCGGCTC<br>R: TAT*ACTAGT*GAAGGCTCGCACATCACCTG
Construction of the *rne*Δ_{CTD} mutant (upstream region)	F: TAT*GAATTC*GACAAGCAGCTGGCGCT<br>R: ATA*GCTAGC*TGACGCACAAAGCGGGTTG
Construction of the *rne*Δ_{CTD} mutant (downstream region)	F: ATA*GCTAGC*TTCGGTCTTGGCCGGCTC<br>R: TAT*ACTAGT*GAAGGCTCGCACATCACCTG
Construction of the Δ*rseA* mutant (upstream region)	F: ATA*ACTAGT*GCGATAACCGTAACGCAGCG<br>R: ATA*GCTAGC*GTATCCTTCGACGCCGGAG
Construction of the Δ*rseA* mutant (downstream region)	F: ATA*GCTAGC*GACTGCCACTCCTTACCAGC<br>R: ATAGAATTCCAACGTGGCGACAAGAAAGC

Construction of chromosomal *hfq*-triple FLAG tag fusion (upstream region)	F: AA*GGATCC*CCAATCCCAATGCCCCGAC<br>R: TA*TTATAA*TCACCGTCATGGTCTTTGTAGTCTTCAGCGGGAACTTCC
Construction of chromosomal *hfq*-triple FLAG tag fusion (downstream region)	F: TA*TTATAA*AGATCATGACATCGATTACAAGGATGACGATGACAAG**TAA**TCGCCTCGCTCCAGC<br>R: AA*GGATCC*CGACCGTGTCCGACAGCACCACCGA
RT-qPCR analysis of *fhaB* expression	F: CAAGGGCGGCAAGGTGA<br>R: ACAGGATGGCGAACAGGCT
RT-qPCR analysis of *ptxA* expression	F: CCAGAACGGATTCACGGC<br>R: CTGCTGCTGGTGGAGACGA
RT-qPCR analysis of *tcfA* expression	F: GACACTTTCTCCTCCCTCGG<br>R: GAAATCCTCCAGAGACATGCC
RT-qPCR analysis of *ompA* expression	F (N primer): CTTGGCGTTGCTATACTGG<br>F (M primer): AACCCTCCAAATTCGCTCT<br>R (L primer): CAGTTGTCCACGGTTTGC
RT-qPCR analysis of *bp0840* expression	F: ATGTACCAGACCCCGTCGTA<br>R: GTCAGCATCCTTGTCGTTCG
RT-qPCR analysis of *bph3* expression	F: CAAACTCAAGCACAAGGCG<br>R: CGATTTCCTCGGGAGTGAT
RT-qPCR analysis of *bp0572* expression	F: GCGCATCTACTTCGAATACC<br>R: CAGCTGCTTGAGCTTGC
RT-qPCR analysis of *bp0663* expression	F: CACCCACGTGAACAAGG<br>R: TCAGTGGTCGAAATTGTGG
RT-qPCR analysis of *bp0913* expression	F: TCTCAAAGGCAAGAAGATCG<br>R: GAAATCCTTGGGACTGAGC
5S_NB probe, detection of 5S rRNA	[Btn]GGGATGGGAAGGAGTGGGACCACC
CT_433_NB probe, detection of CT_433 sRNA	AAAGGAGGAGGGTGGAGGAGACAA[Btn]
CT_521_NB probe, detection of CT_521 sRNA	GGGAGGGGAAAGAGGAGAAGCCG[Btn]

RgtA_NB probe, detection of RgtA sRNA	[Btn]GCCGCCTTTCAAAGCACAGG
ompA_NB probe, detection of the ompA transcript	GCTTCTGGTTGTAGGCTTCGGTGC[Btn]
CT_532NB probe, detection of CT_532 sRNA	[Btn]GCCCGAATTCCAGCTCTGCTCT
bp2374_5end probe, detection of *bp2374* RNA	CGGAAGGTATTCGGGTTCATCGCG[Btn]
bp2374_3end probe, detection of *bp2374* RNA	GAGCGGGTCTTAAAAGGGGCTTTGCAGC[Btn]

*F, forward primer; R, reverse primer; sequences shown in italics indicate restriction enzyme recognition sites added for cloning purposes, the sequence corresponding to triple FLAG tag is underlined, stop codon (TAA) of hfq gene is shown in bold; [Btn], probes are labeled by biotin at 5´ or 3´ end.

Kumar et al. Supplementary Figure S1

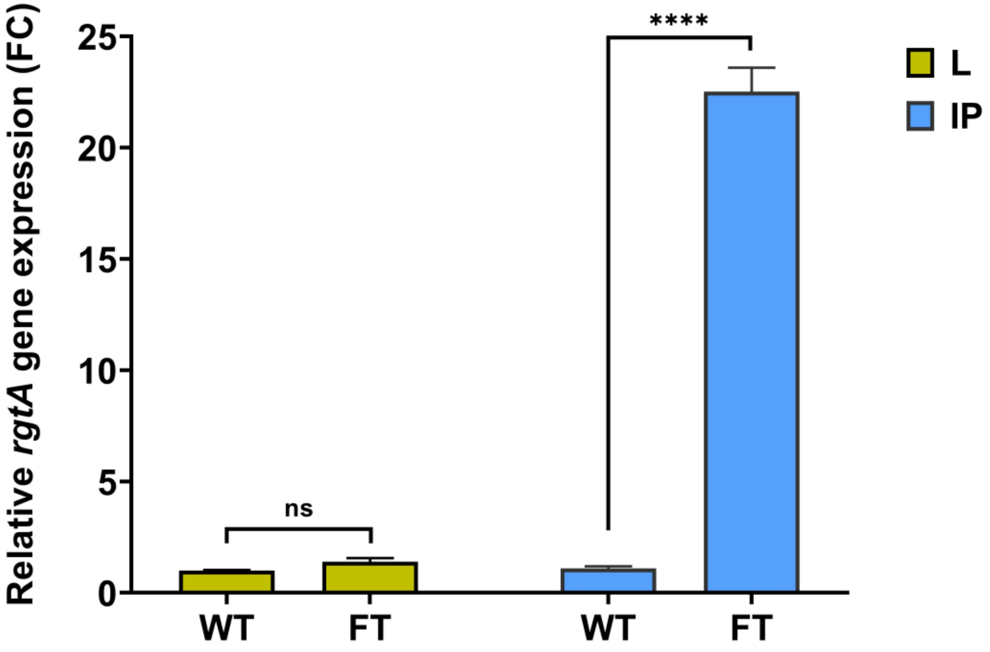

Supplementary Figure S1. The relative expression of RgtA sRNA was determined in RNA samples isolated from *hfq*-WT (WT) and *hfq*::3xFLAG (FT) cells. The relative expression of *rgtA* gene was determined with the total RNA isolated from cell lysates (L; yellow bars) and with RNA immunoprecipitated by the wt Hfq and FLAG-tagged Hfq on magnetic beads (IP; blue bars). The relative expression of RgtA in RNA isolated from the total lysate of *hfq*-WT was set to 1. Fold change values are means (bars) ± standard deviations (error bars) from three biological replicate experiments. Statistical analysis was performed using the two-way ANOVA test for multiple comparisons (Sidak´s test); ns, p-value > 0.05; ****, p-value < 0.0001.

Kumar et al. Supplementary Figure S2

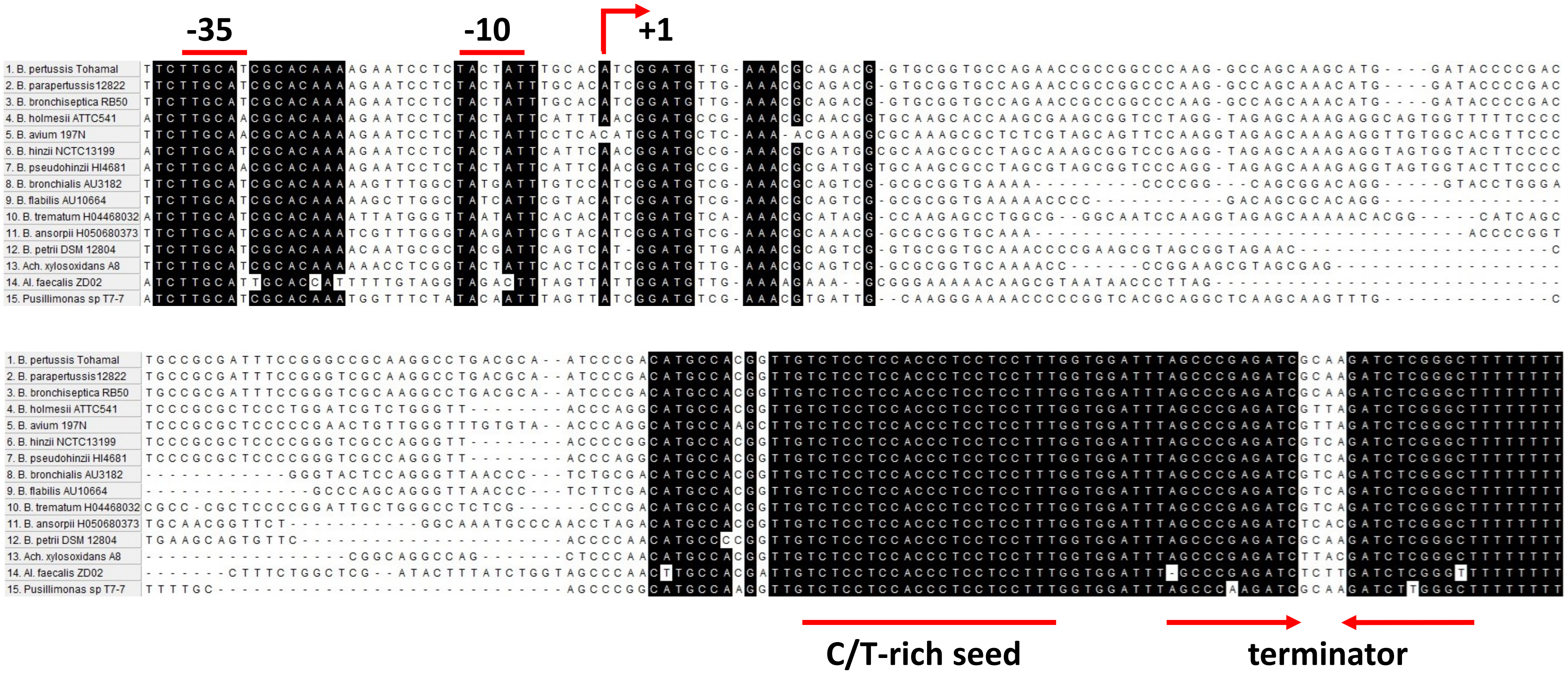

Supplementary Figure S2. Conservation of CT_433 sRNA in bordetellae and related bacteria. The DNA sequence corresponding to the *ct_433* locus of the *B. pertussis* Tohama I strain, and the genomic sequences of the indicated strains were aligned with the MUSCLE algorithm built in the MEGA software. The black background indicates the nucleotides that are highly conserved (> 90% conservation). The promoter region including the plausible -35 and -10 sequences (underlined), the transcription start site of CT_433 (+1; bent arrow), the C/T-rich conserved region and the terminator are shown in red.

Kumar et al. Supplementary Figure S3

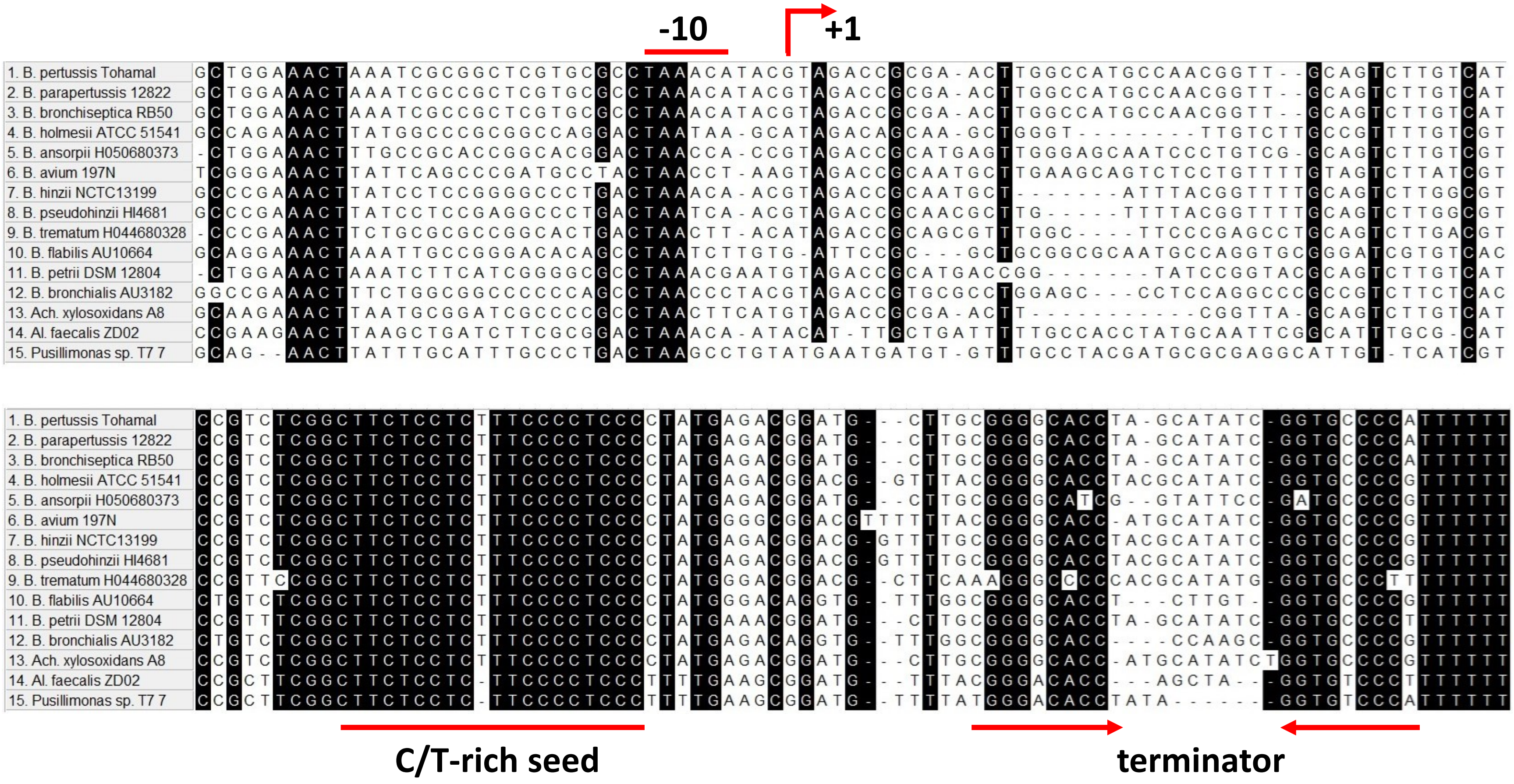

Supplementary Figure S3. Conservation of CT_521 sRNA in bordetellae and related bacteria. The DNA sequence corresponding to the *ct_521* locus of the *B. pertussis* Tohama I strain, and the genomic sequences of the indicated strains were aligned with the MUSCLE algorithm built in the MEGA software. The black background depicts the nucleotides that are highly conserved (> 90% conservation). The promoter region including the plausible -35 and -10 sequences (underlined), the transcription start site of CT_521 (+1; bent arrow), the C/T-rich conserved region and the terminator are shown in red.

Kumar et al. Figure S4

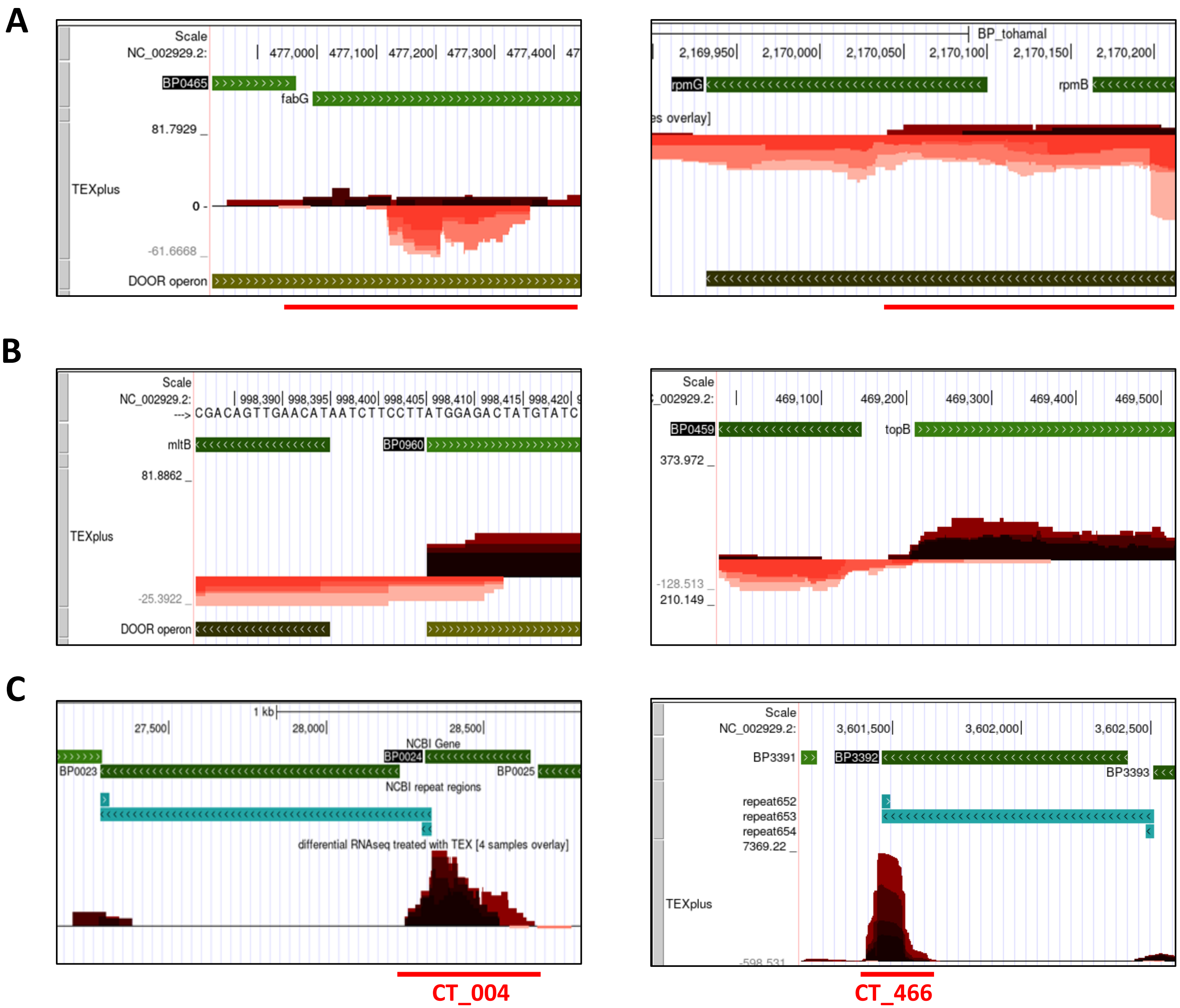

Supplementary Figure S4. RIL-seq identifies S-chimeras resulting from operonic structures, overlapping transcription and IS*481*-derived sRNAs **(A)** Web browser-derived screenshot of the differential RNA-seq normalized dataset showing the operonic genomic regions between the *bp0465* and *bp0466* (*fabG*) genes (left panel) and the *bp2050* (*rpmG*) and *bp2051* (*rpmB*) genes (right panel). Graphs display the sequencing depth of the positive (dark red) and negative (light red) strands obtained with the TEX-treated library. The different color intensities represent the different library replicates. The gene annotations are depicted as green arrows. Red bars below the images indicate the genomic position of an antisense RNA that spans both genes. **(B)** Web browser-derived screenshot of the differential RNA-seq normalized dataset showing overlapping head-to-head transcription in the genomic regions between the *bp0959* (*mltB*) and *bp0960* genes (left panel) and the *bp0459* and *bp0460* (*topB*) genes (right panel). Graphs display the sequencing depth of the positive (dark red) and negative (light red) strands obtained with the TEX-treated library. The different color intensities represent the different library replicates. The gene annotations are depicted as green arrows. **(C)** Web browser-derived screenshot of the differential RNA-seq normalized dataset showing the genomic regions between the *bp0023* and *bp0024* genes including the CT_004 sRNA (left panel) and the *bp3391* and *bp3392* genes including the CT_466 sRNA (right panel). Graphs display the sequencing depth of the positive and negative strands obtained with the TEX-treated library. The different color intensities represent the different library replicates. The gene annotations are depicted as green arrows, transposase genes and direct repeats are shown in cyan. Red bars below the images indicate the genomic position of CT_004 and CT_466 sRNAs.

Kumar et al. Supplementary Figure S5

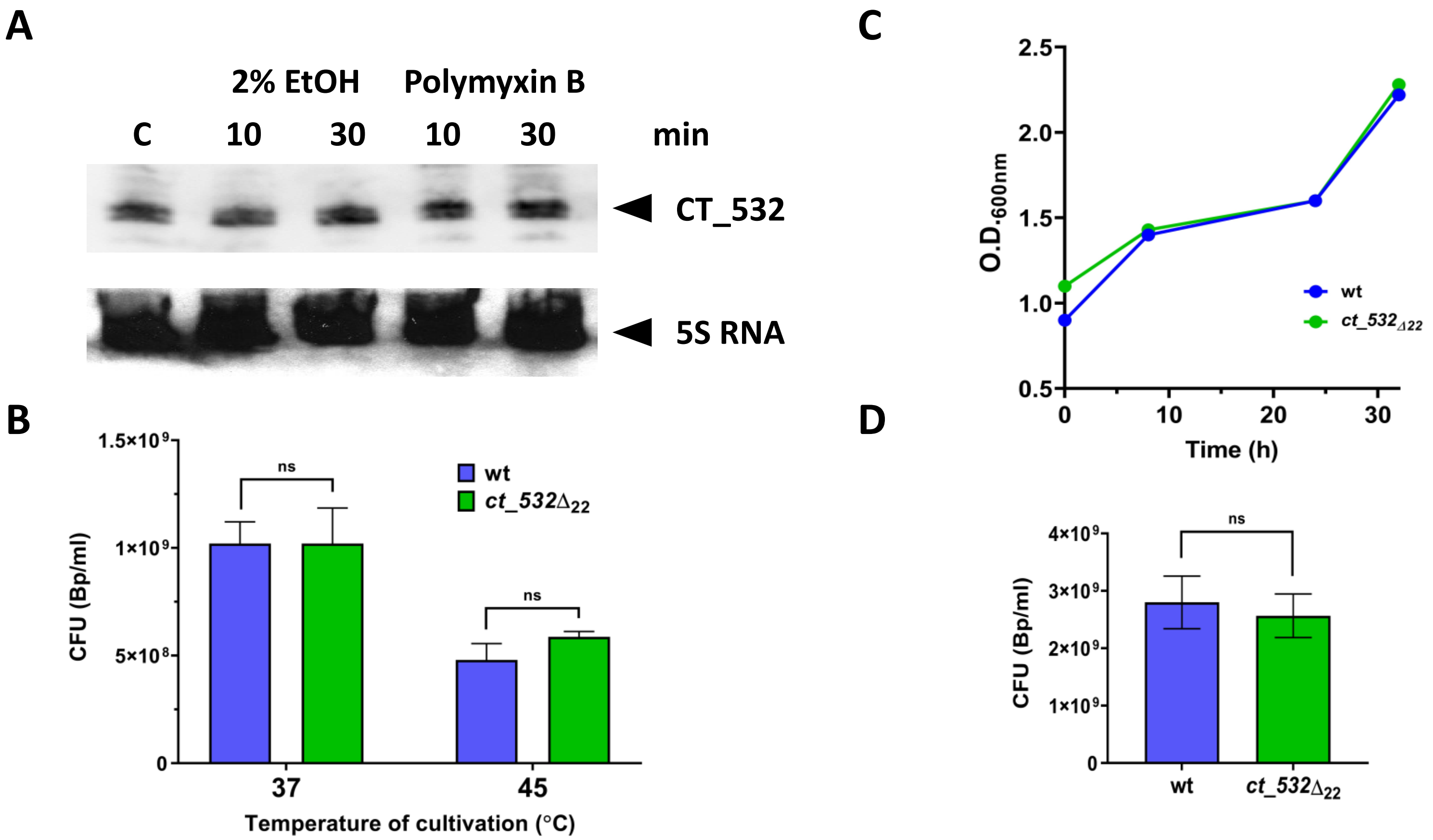

Supplementary Figure S5. The effects of heat and cold shocks on CT_532 expression and survival of wt and *ct_532*$_{\Delta 22}$ cells. **(A)** *B. pertussis* cells were grown at 37 °C to mid exponential phase and then stressed by treatment with 2% ethanol or with 5 µg/ml polymyxin B to induce membrane stress. Total RNA isolated from cells 10 min and 30 min after the treatment was used to detect CT_532 and 5S RNAs. Untreated cells were used as control. **(B)** wt and *ct_532*$_{\Delta 22}$ cells were grown overnight at 37 °C to early stationary phase. Then, the cells were transferred and incubated at 45 °C for 60 min to induce heat shock conditions. Immediately after the stress cells were serially diluted and plated for CFU counting. The graph shows the CFUs for treated and untreated cells. CFU values are means (bars) ± standard deviations (error bars) from three biological replicate experiments. Statistical analysis was performed using the two-way ANOVA test for multiple comparisons (Sidak's test); ns, p-value > 0.05 **(C)** wt and *ct_532*$_{\Delta 22}$ cells were grown overnight at 37 °C to an OD600 of 1 and then were transferred to 20 °C. The density of the cultures was monitored for 32 hours. **(D)** Cells grown at 20 °C (see panel C) to an OD600 of 1.5 (time point 24 h) were serially diluted and plated for CFU counting. CFU values are means (bars) ± standard deviations (error bars) from three biological replicate experiments. Statistical analysis was performed using the unpaired t-test; ns, p-value > 0.05.
